# Supplementary material for: Newly discovered and conserved role of IgM against viral infection in an early vertebrate
Source: eLife. 2025 Sep 4;14:RP104465. doi: 10.7554/eLife.104465 (PMC12410970; doi:10.7554/eLife.104465)
Supplement: Figure 3—figure supplement 1—source data 1. [file elife-104465-fig3-figsupp1-data1.pdf]

Figure 3–figure supplement 1A

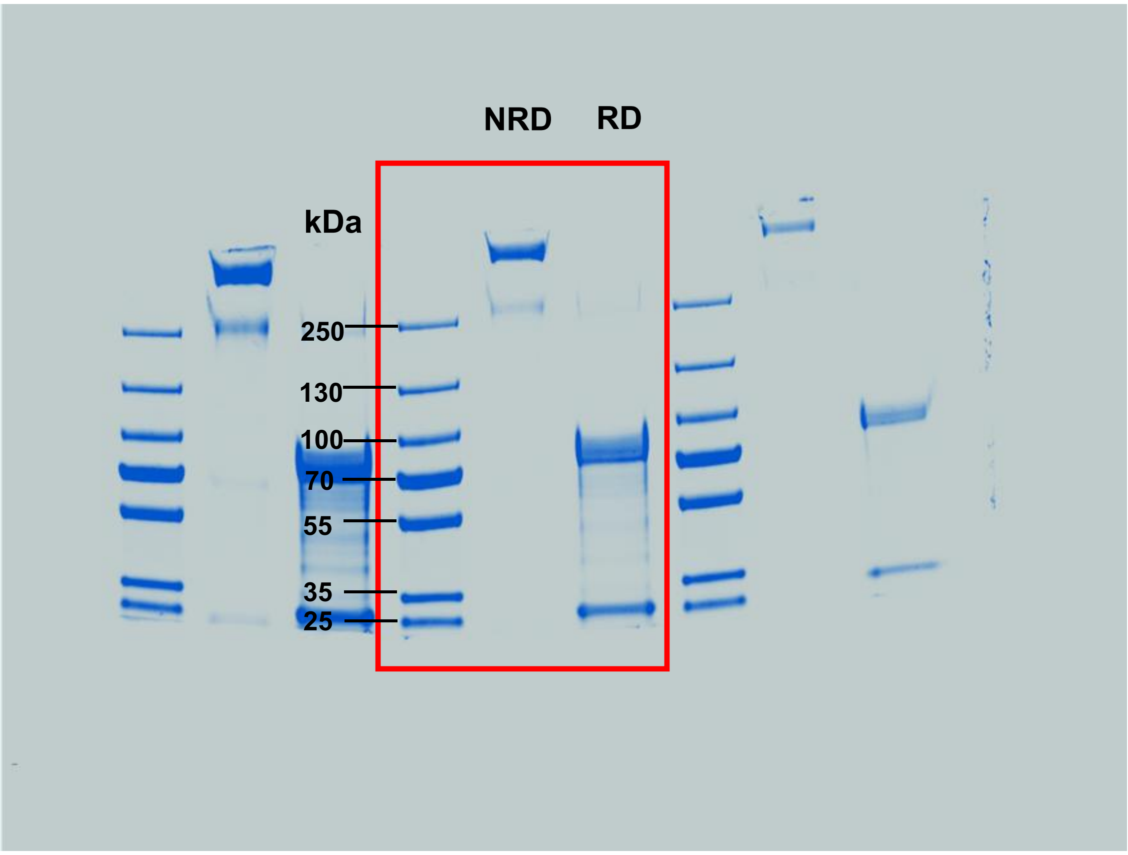

Figure 3–figure supplement 1 Original membranes corresponding to Figure 3–figure supplement 1A. Molecular weight markers were employed. The areas highlighted by red boxes are used in this result figure.
