# Supplementary figures and images for: Newly discovered and conserved role of IgM against viral infection in an early vertebrate
Source: eLife. 2025 Sep 4;14:RP104465. doi: 10.7554/eLife.104465 (PMC12410970; doi:10.7554/eLife.104465)

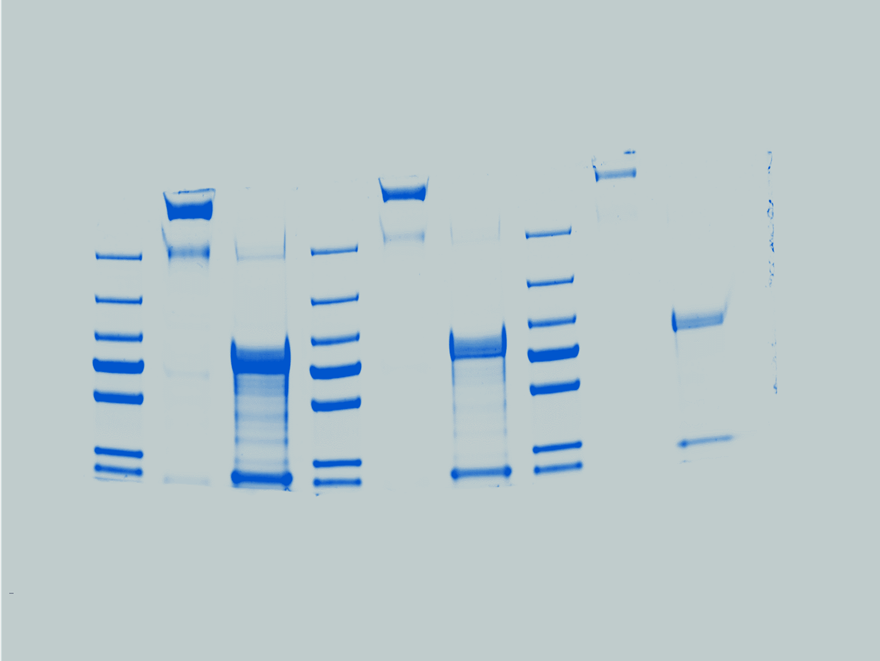

Supplement: Figure 3—figure supplement 1—source data 2. [file elife-104465-fig3-figsupp1-data2.zip › Figure 3-figure supplement 1-source data 2/Figure 3-figure supplement 1A NRD RD.png]

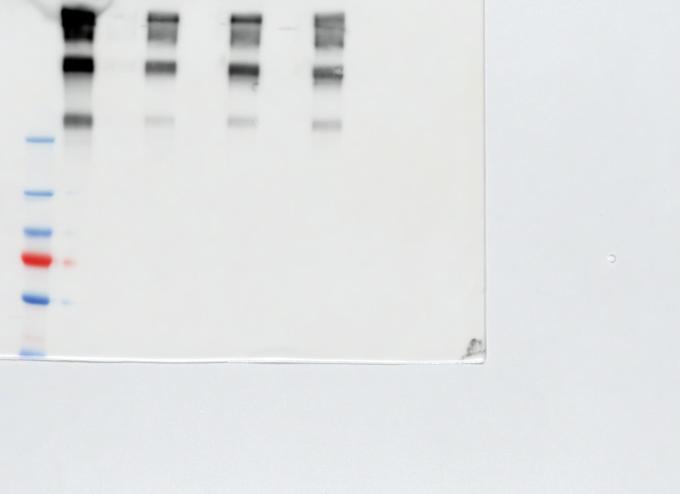

Supplement: Figure 3—figure supplement 2—source data 2. [file elife-104465-fig3-figsupp2-data2.zip › Figure 3-figure supplement 2-source data 2/Figure 3-figure supplement 2A NRD.jpg]

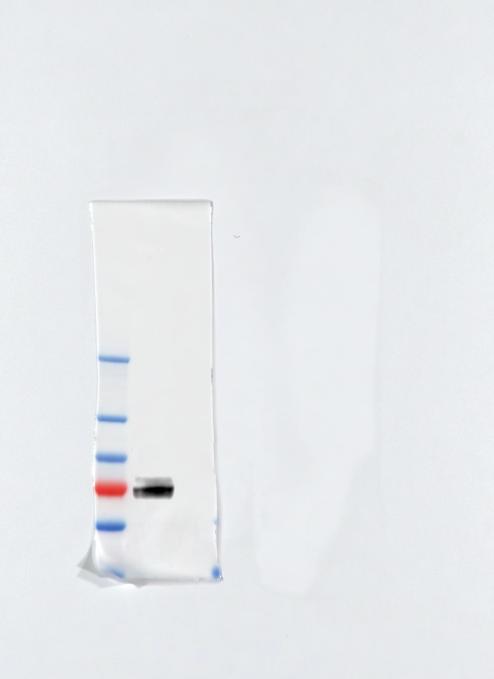

Supplement: Figure 3—figure supplement 2—source data 2. [file elife-104465-fig3-figsupp2-data2.zip › Figure 3-figure supplement 2-source data 2/Figure 3-figure supplement 2A RD.jpg]

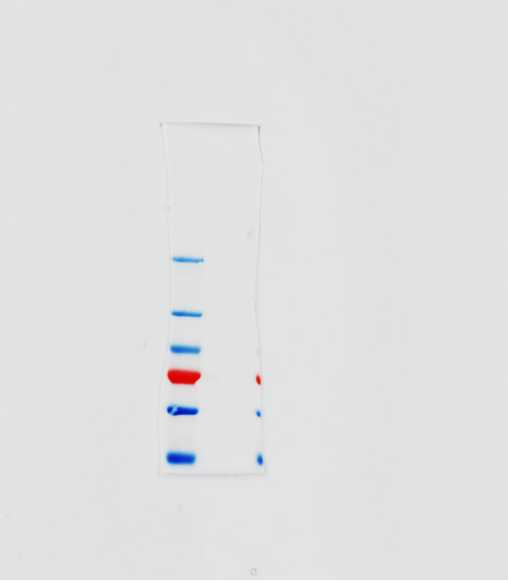

Supplement: Figure 3—figure supplement 2—source data 2. [file elife-104465-fig3-figsupp2-data2.zip › Figure 3-figure supplement 2-source data 2/Figure 3-figure supplement 2B NRD.jpg]

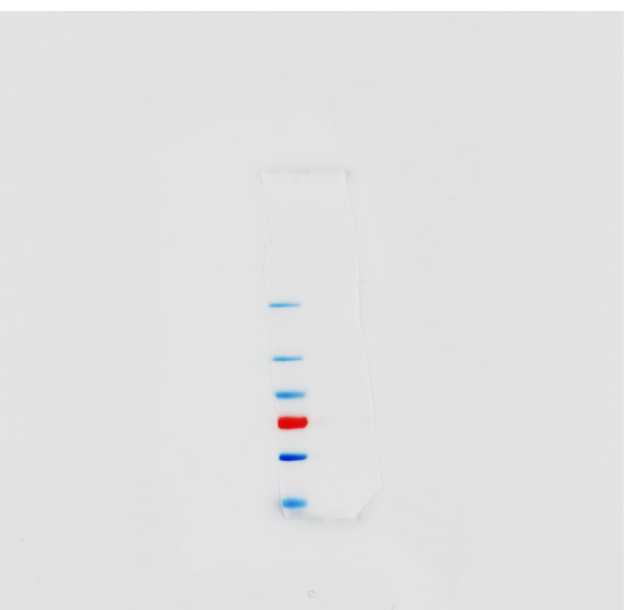

Supplement: Figure 3—figure supplement 2—source data 2. [file elife-104465-fig3-figsupp2-data2.zip › Figure 3-figure supplement 2-source data 2/Figure 3-figure supplement 2B RD.jpg]

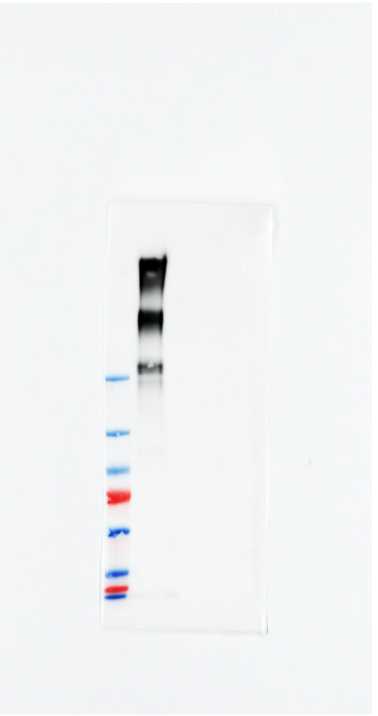

Supplement: Figure 3—figure supplement 2—source data 2. [file elife-104465-fig3-figsupp2-data2.zip › Figure 3-figure supplement 2-source data 2/Figure 3-figure supplement 2C NRD.jpg]

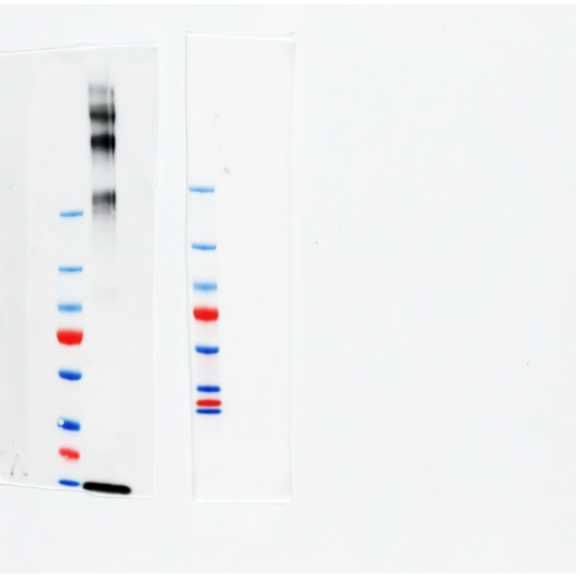

Supplement: Figure 3—figure supplement 2—source data 2. [file elife-104465-fig3-figsupp2-data2.zip › Figure 3-figure supplement 2-source data 2/Figure 3-figure supplement 2C RD.jpg]

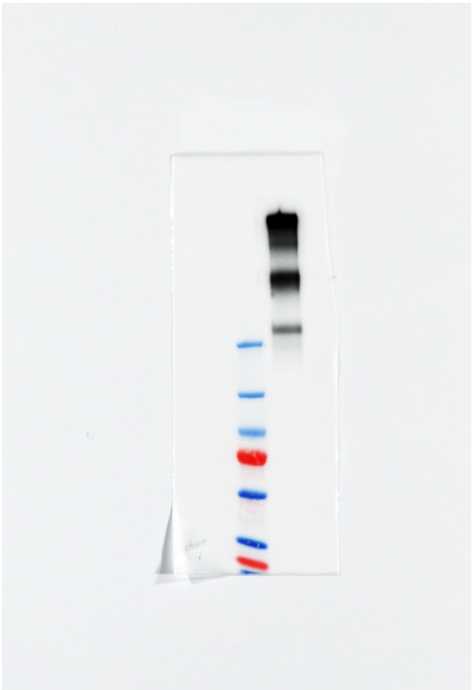

Supplement: Figure 3—figure supplement 2—source data 2. [file elife-104465-fig3-figsupp2-data2.zip › Figure 3-figure supplement 2-source data 2/Figure 3-figure supplement 2D NRD.jpg]

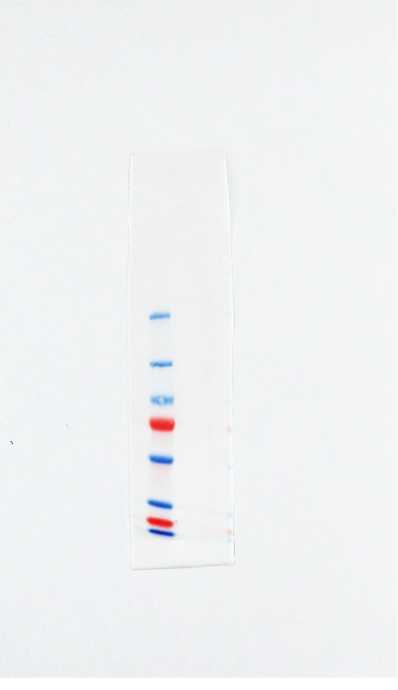

Supplement: Figure 3—figure supplement 2—source data 2. [file elife-104465-fig3-figsupp2-data2.zip › Figure 3-figure supplement 2-source data 2/Figure 3-figure supplement 2D RD.jpg]

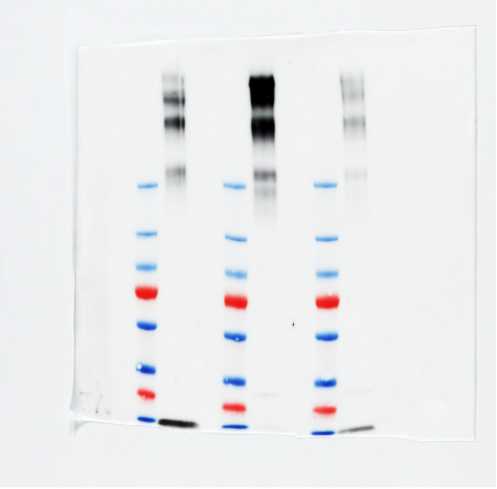

Supplement: Figure 3—figure supplement 2—source data 2. [file elife-104465-fig3-figsupp2-data2.zip › Figure 3-figure supplement 2-source data 2/Figure 3-figure supplement 2E NRD.jpg]

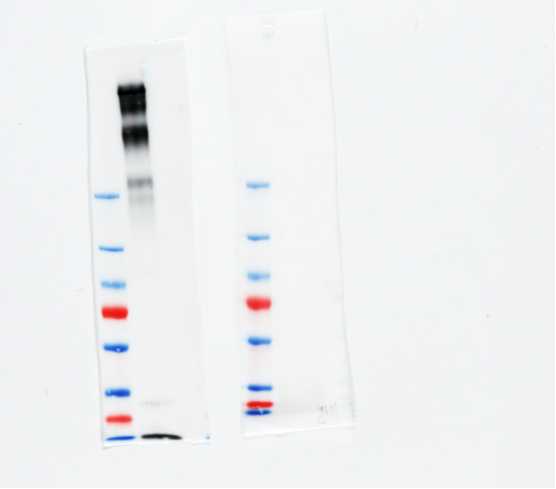

Supplement: Figure 3—figure supplement 2—source data 2. [file elife-104465-fig3-figsupp2-data2.zip › Figure 3-figure supplement 2-source data 2/Figure 3-figure supplement 2E RD.jpg]

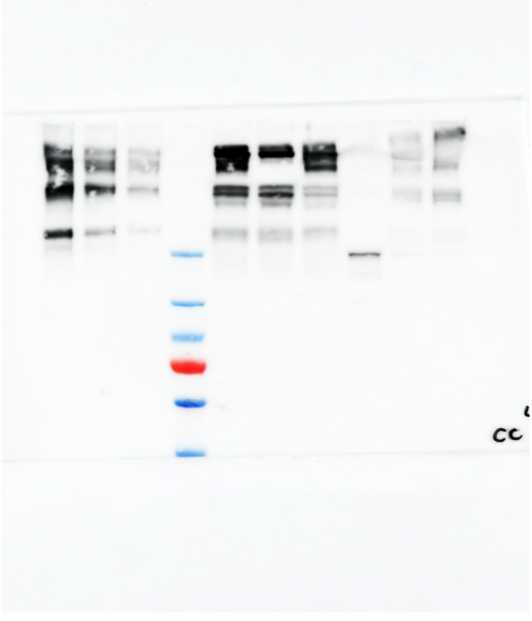

Supplement: Figure 3—figure supplement 2—source data 2. [file elife-104465-fig3-figsupp2-data2.zip › Figure 3-figure supplement 2-source data 2/Figure 3-figure supplement 2F NRD.jpg]

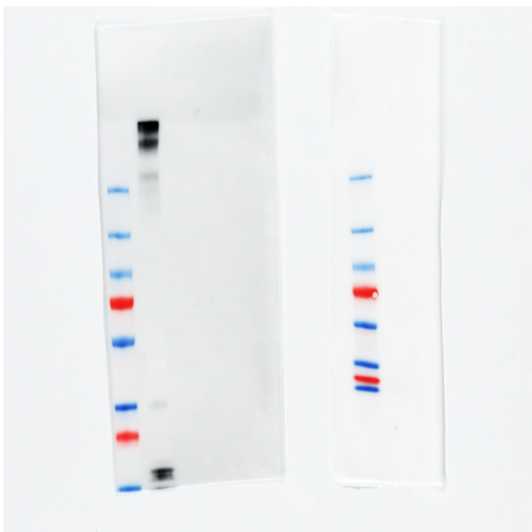

Supplement: Figure 3—figure supplement 2—source data 2. [file elife-104465-fig3-figsupp2-data2.zip › Figure 3-figure supplement 2-source data 2/Figure 3-figure supplement 2F RD.jpg]

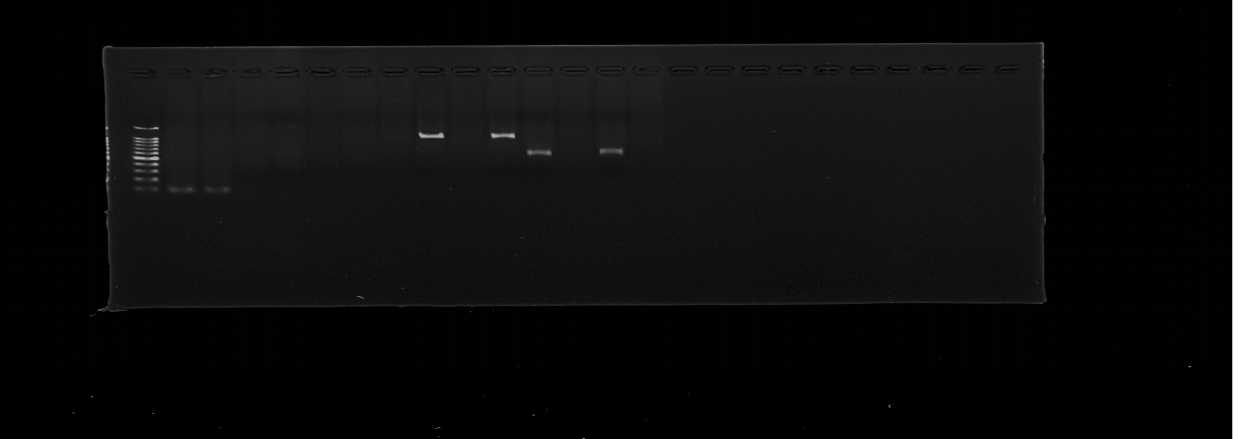

Supplement: Figure 3—figure supplement 2—source data 2. [file elife-104465-fig3-figsupp2-data2.zip › Figure 3-figure supplement 2-source data 2/Figure 3-figure supplement 2H IgD IgT.jpg]

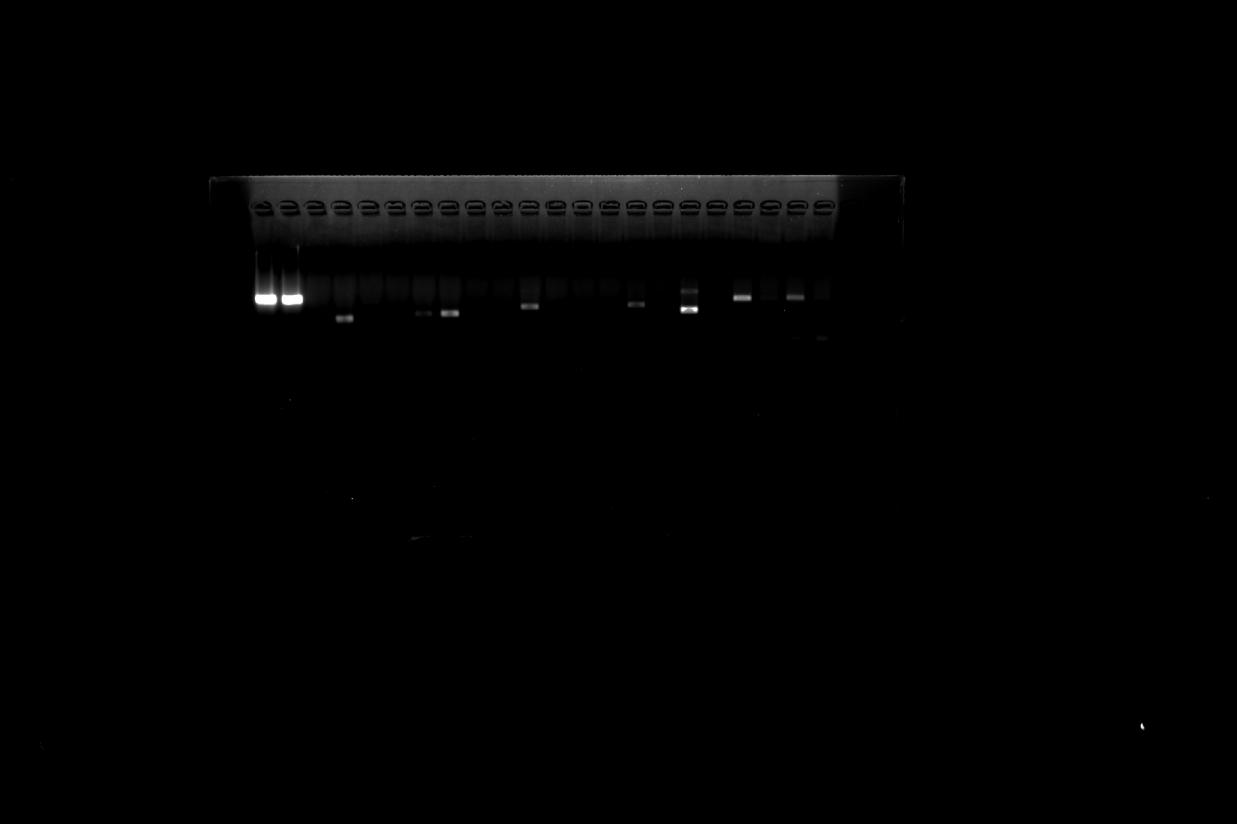

Supplement: Figure 3—figure supplement 2—source data 2. [file elife-104465-fig3-figsupp2-data2.zip › Figure 3-figure supplement 2-source data 2/Figure 3-figure supplement 2H β-actin IgM CD3 CD3 TCRα.jpg]

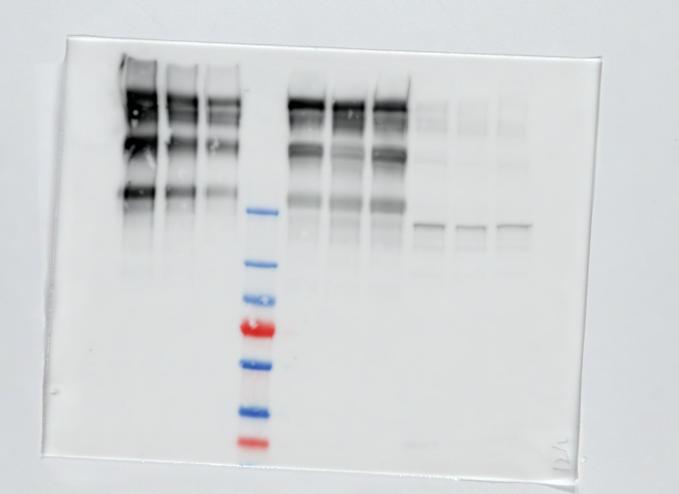

Supplement: Figure 5—figure supplement 2—source data 2. [file elife-104465-fig5-figsupp2-data2.zip › Figure 5-figure supplement 2-source data 2/Figure 5-figure supplement 2A Day1.jpg]

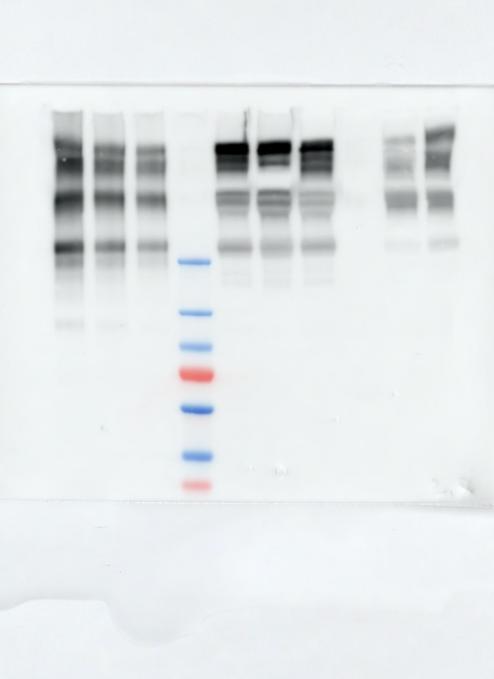

Supplement: Figure 5—figure supplement 2—source data 2. [file elife-104465-fig5-figsupp2-data2.zip › Figure 5-figure supplement 2-source data 2/Figure 5-figure supplement 2A Day10.jpg]

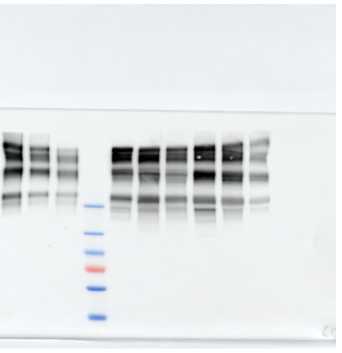

Supplement: Figure 5—figure supplement 2—source data 2. [file elife-104465-fig5-figsupp2-data2.zip › Figure 5-figure supplement 2-source data 2/Figure 5-figure supplement 2A Day14.jpg]

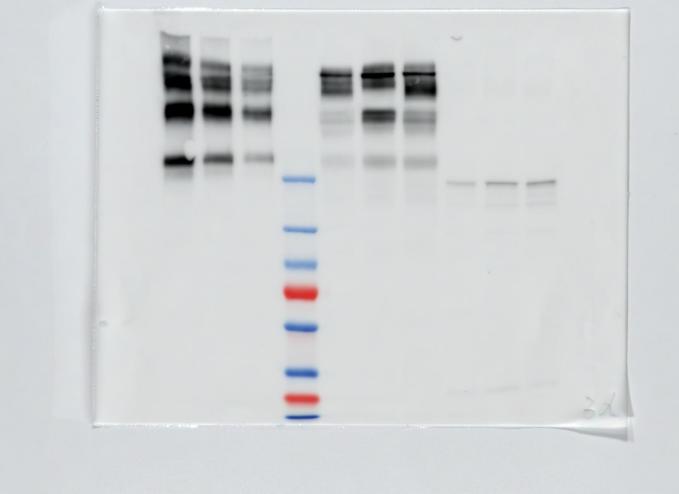

Supplement: Figure 5—figure supplement 2—source data 2. [file elife-104465-fig5-figsupp2-data2.zip › Figure 5-figure supplement 2-source data 2/Figure 5-figure supplement 2A Day2.jpg]

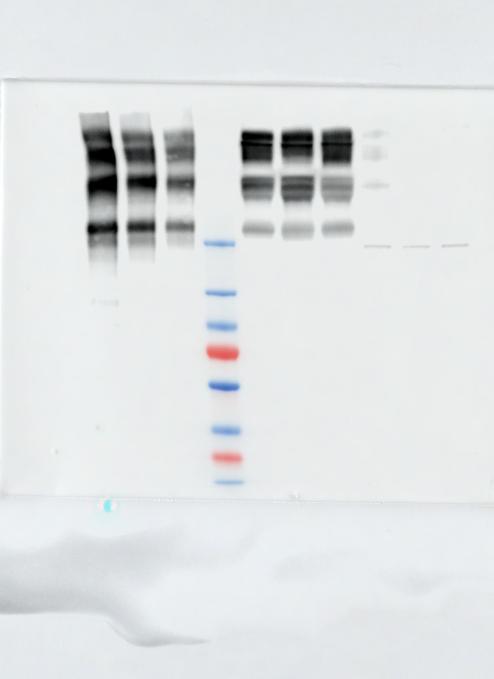

Supplement: Figure 5—figure supplement 2—source data 2. [file elife-104465-fig5-figsupp2-data2.zip › Figure 5-figure supplement 2-source data 2/Figure 5-figure supplement 2A Day3.jpg]

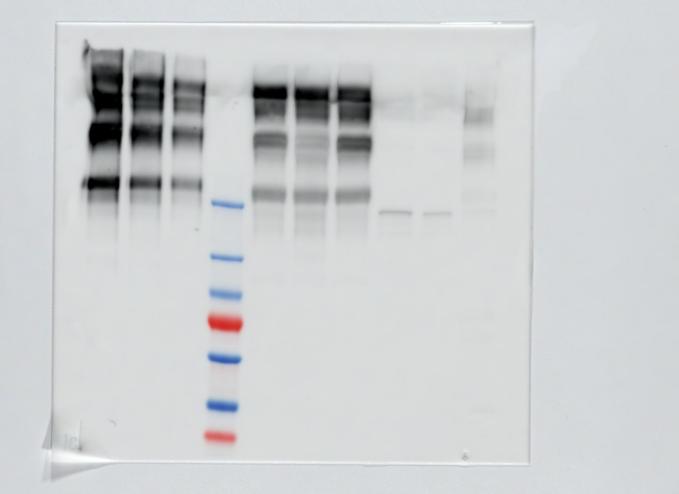

Supplement: Figure 5—figure supplement 2—source data 2. [file elife-104465-fig5-figsupp2-data2.zip › Figure 5-figure supplement 2-source data 2/Figure 5-figure supplement 2A Day4.jpg]

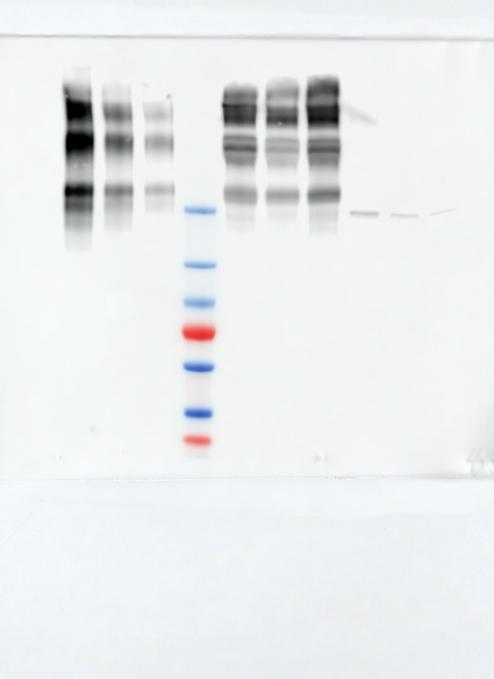

Supplement: Figure 5—figure supplement 2—source data 2. [file elife-104465-fig5-figsupp2-data2.zip › Figure 5-figure supplement 2-source data 2/Figure 5-figure supplement 2A Day5.jpg]

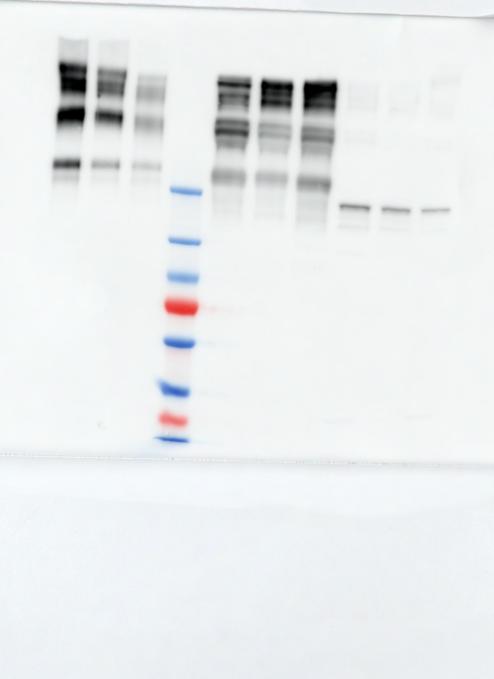

Supplement: Figure 5—figure supplement 2—source data 2. [file elife-104465-fig5-figsupp2-data2.zip › Figure 5-figure supplement 2-source data 2/Figure 5-figure supplement 2A Day7.jpg]

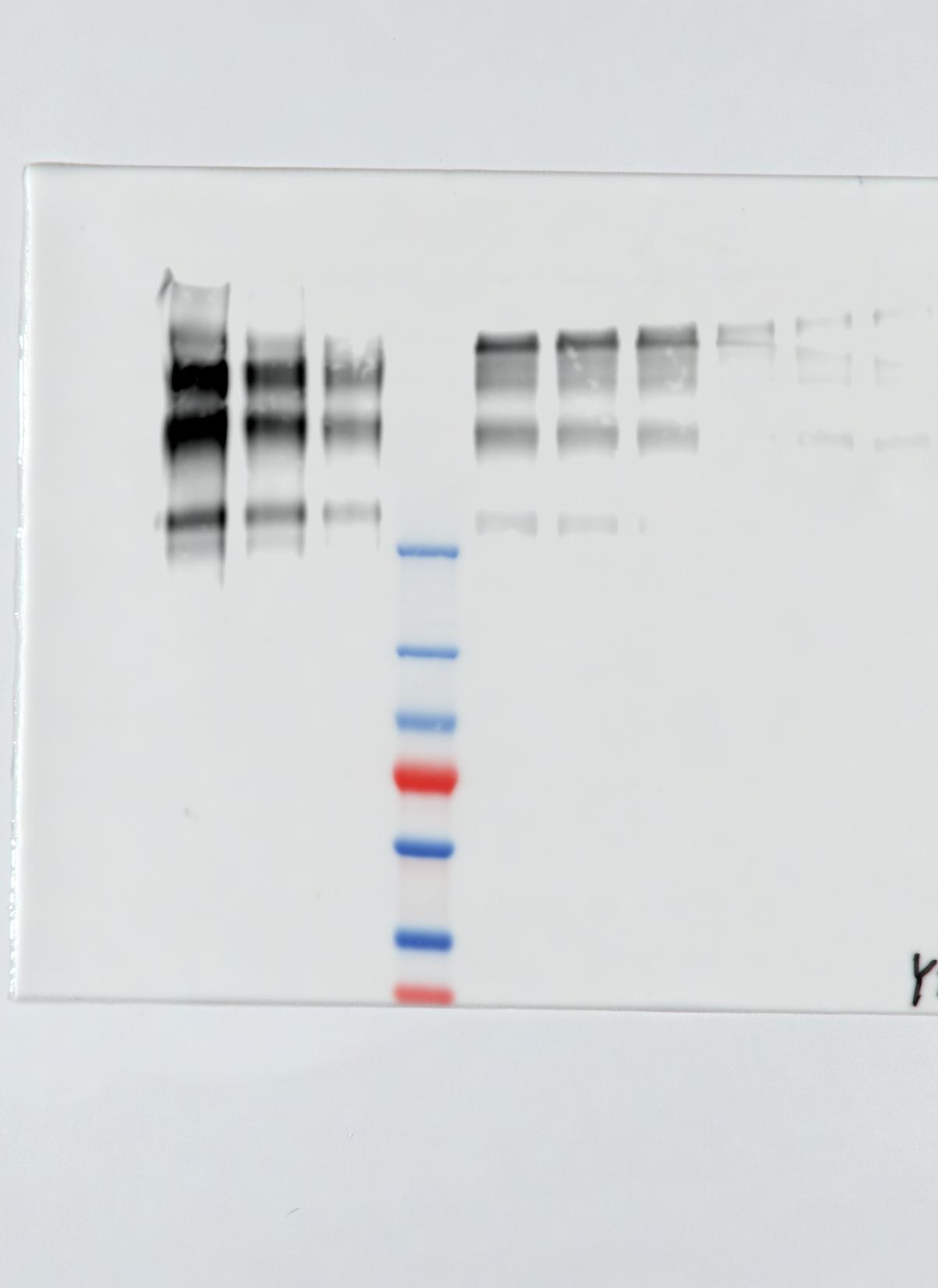

Supplement: Figure 5—figure supplement 2—source data 2. [file elife-104465-fig5-figsupp2-data2.zip › Figure 5-figure supplement 2-source data 2/Figure 5-figure supplement 2B Day1.jpg]

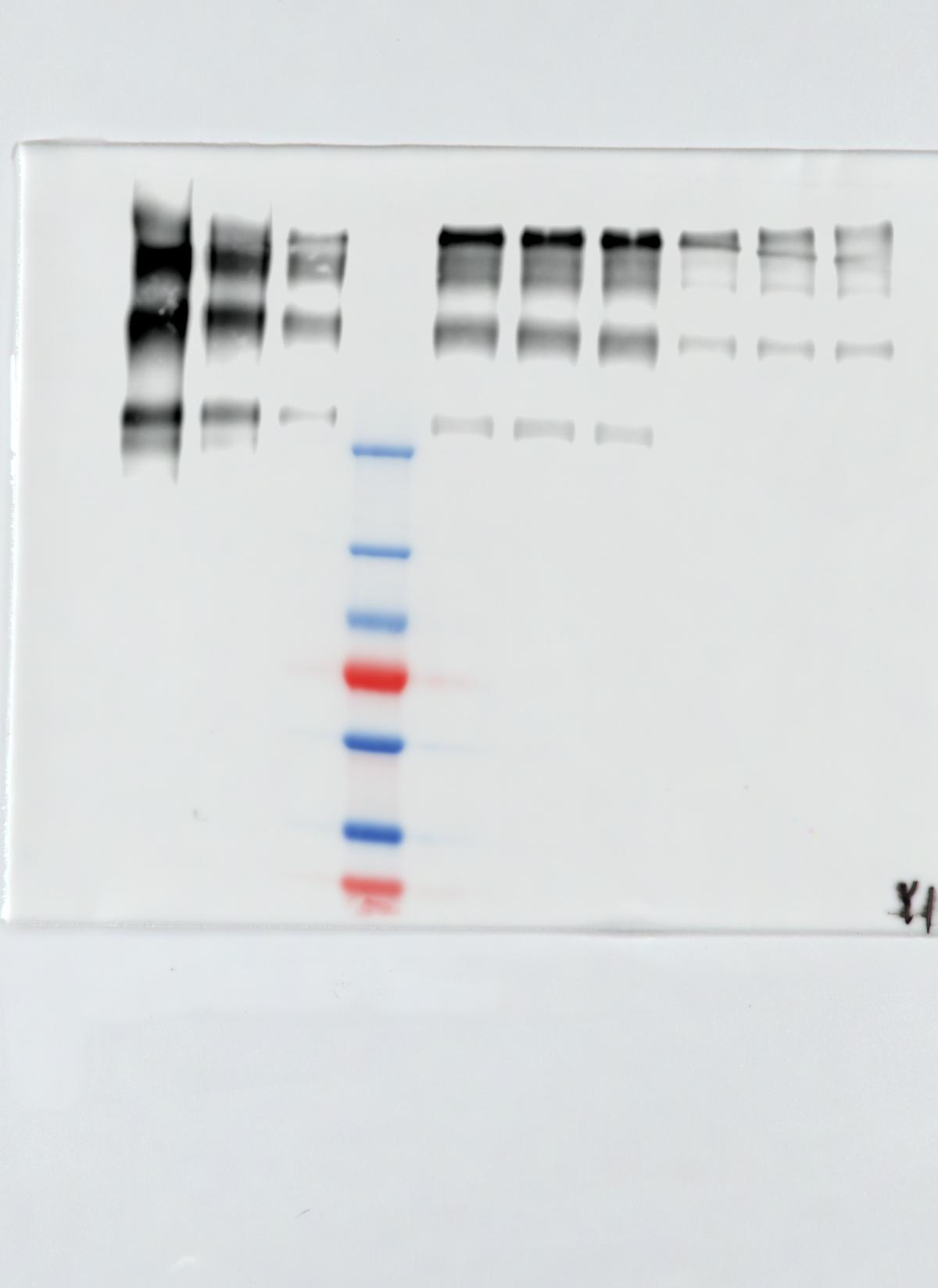

Supplement: Figure 5—figure supplement 2—source data 2. [file elife-104465-fig5-figsupp2-data2.zip › Figure 5-figure supplement 2-source data 2/Figure 5-figure supplement 2B Day10.jpg]

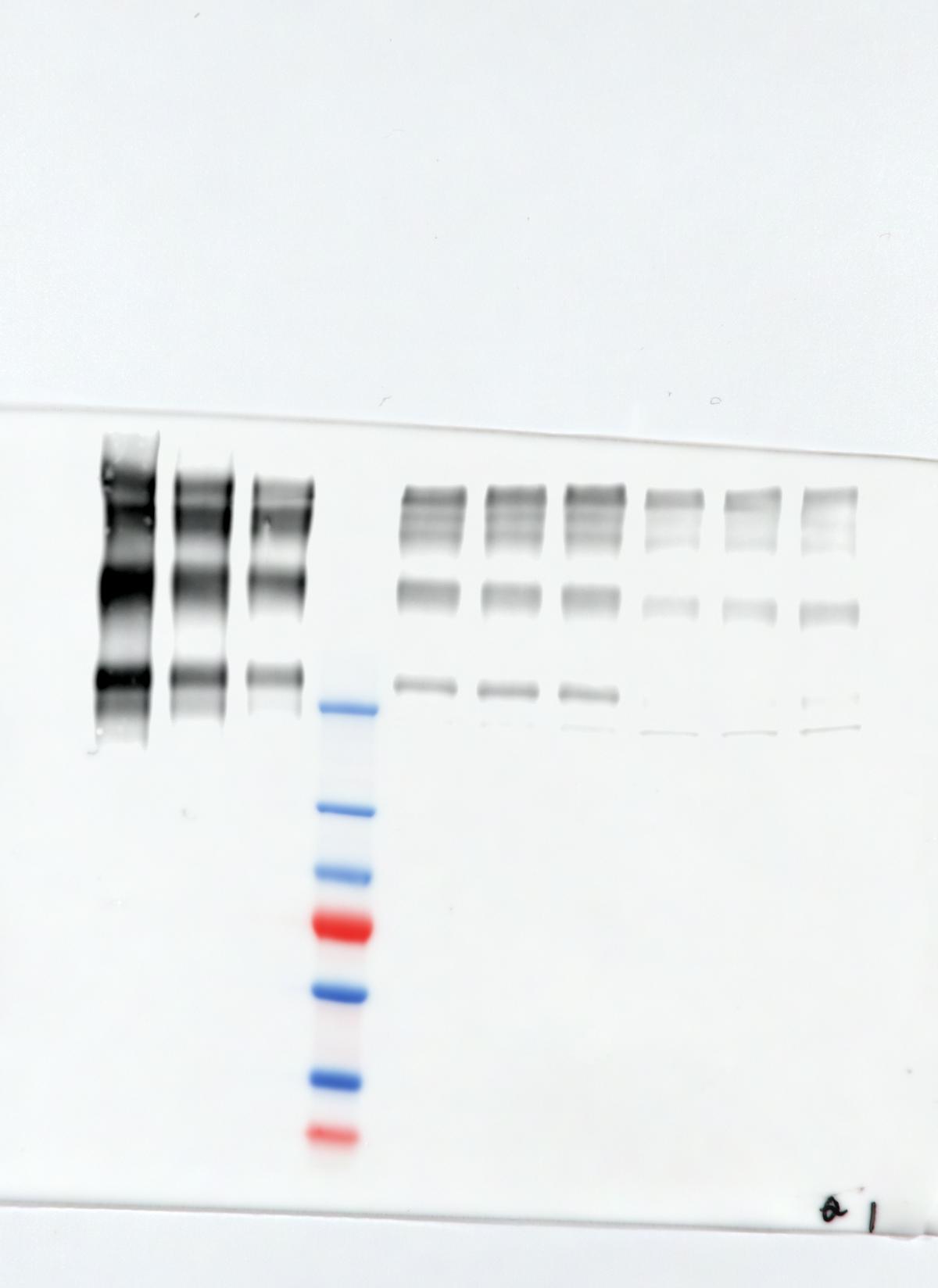

Supplement: Figure 5—figure supplement 2—source data 2. [file elife-104465-fig5-figsupp2-data2.zip › Figure 5-figure supplement 2-source data 2/Figure 5-figure supplement 2B Day14.jpg]

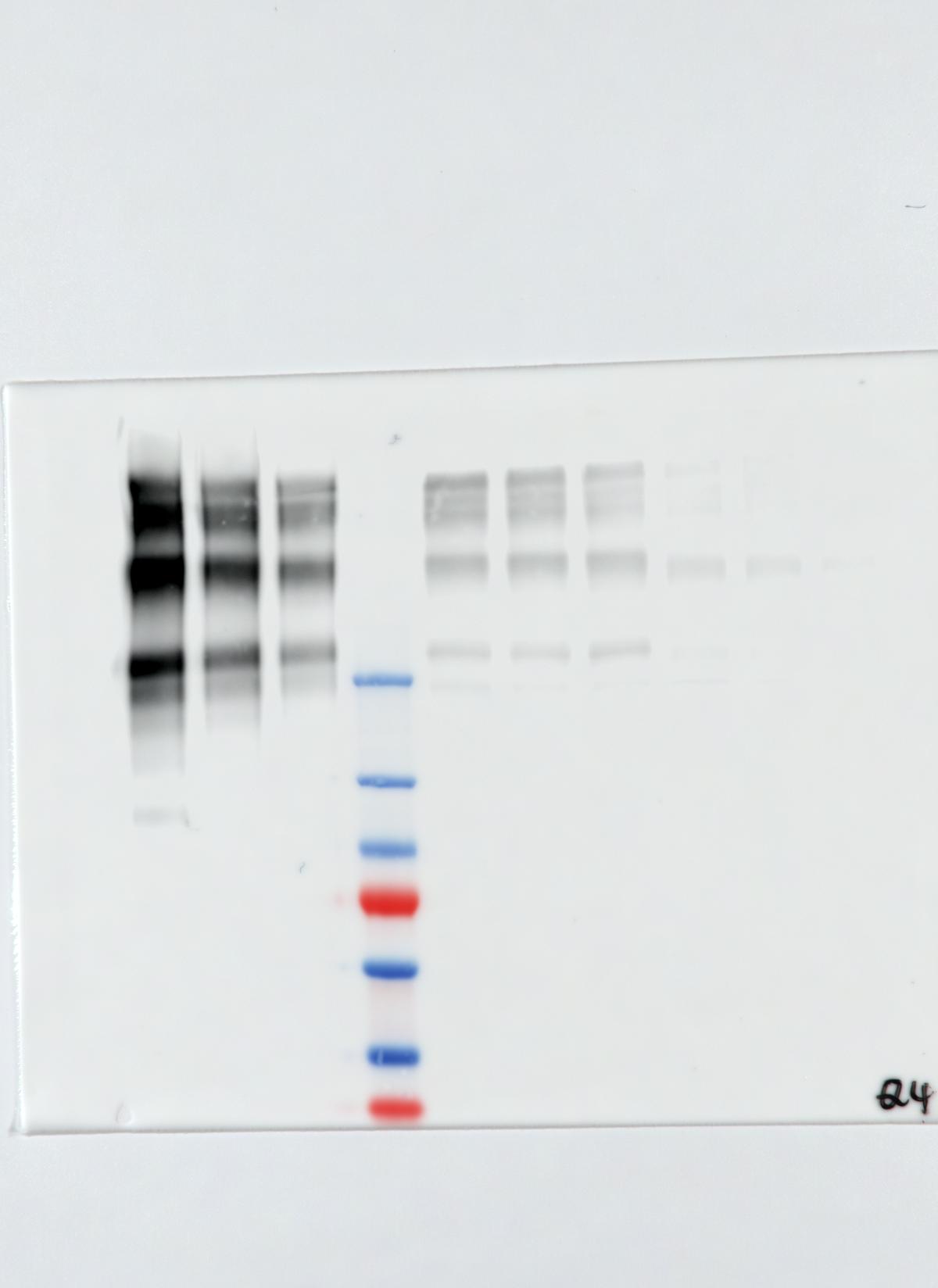

Supplement: Figure 5—figure supplement 2—source data 2. [file elife-104465-fig5-figsupp2-data2.zip › Figure 5-figure supplement 2-source data 2/Figure 5-figure supplement 2B Day2.jpg]

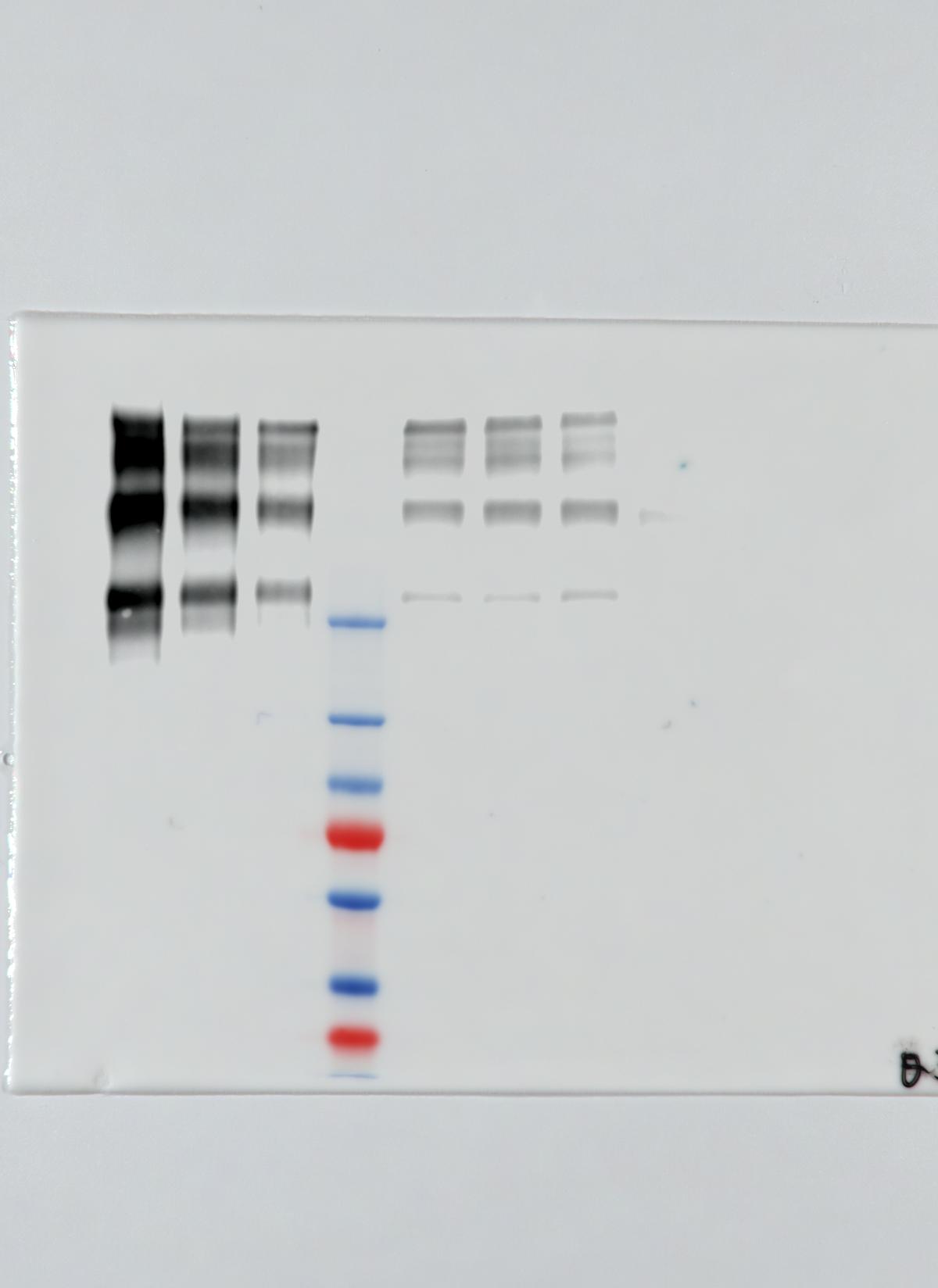

Supplement: Figure 5—figure supplement 2—source data 2. [file elife-104465-fig5-figsupp2-data2.zip › Figure 5-figure supplement 2-source data 2/Figure 5-figure supplement 2B Day3.jpg]

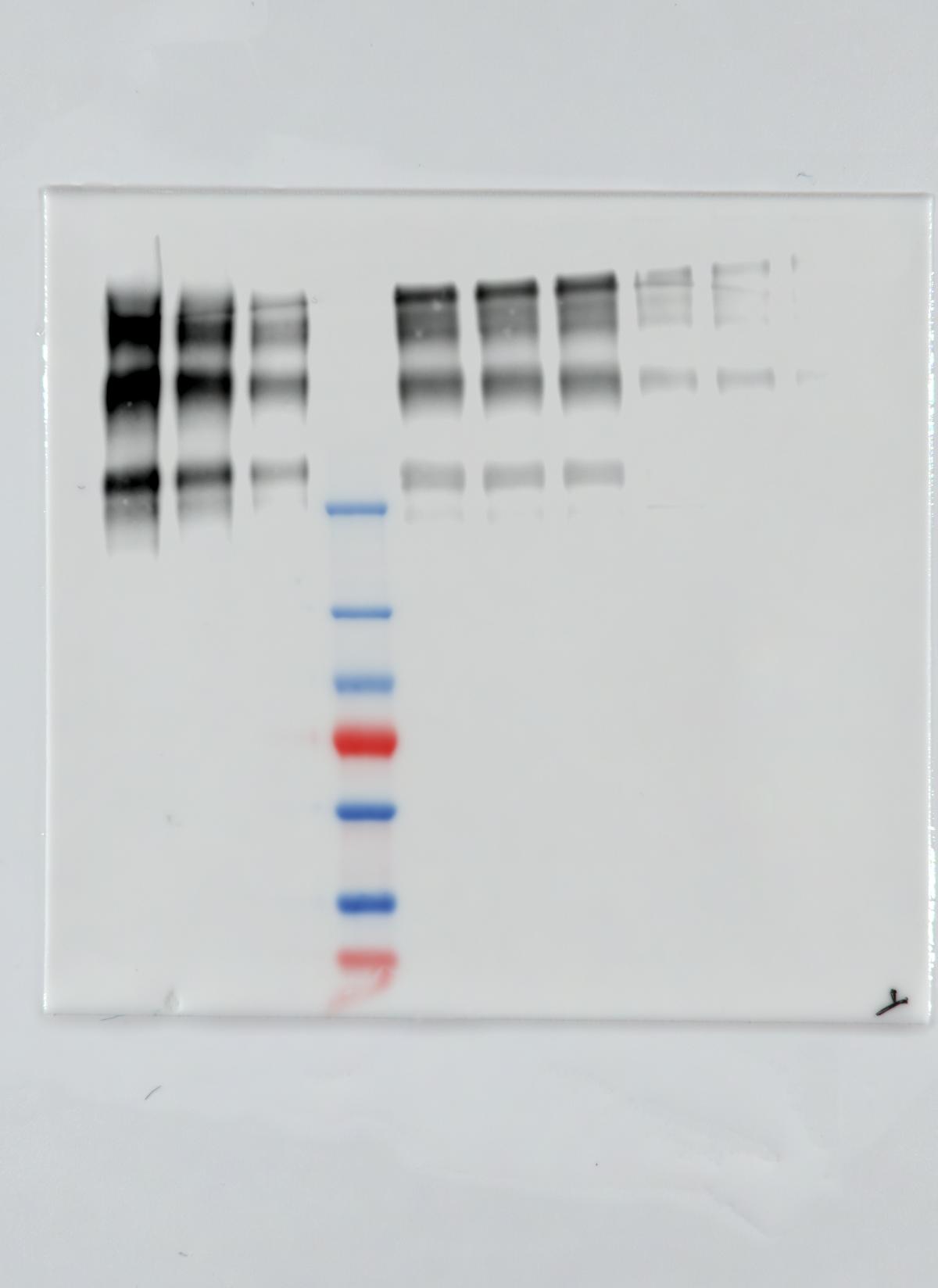

Supplement: Figure 5—figure supplement 2—source data 2. [file elife-104465-fig5-figsupp2-data2.zip › Figure 5-figure supplement 2-source data 2/Figure 5-figure supplement 2B Day4.jpg]

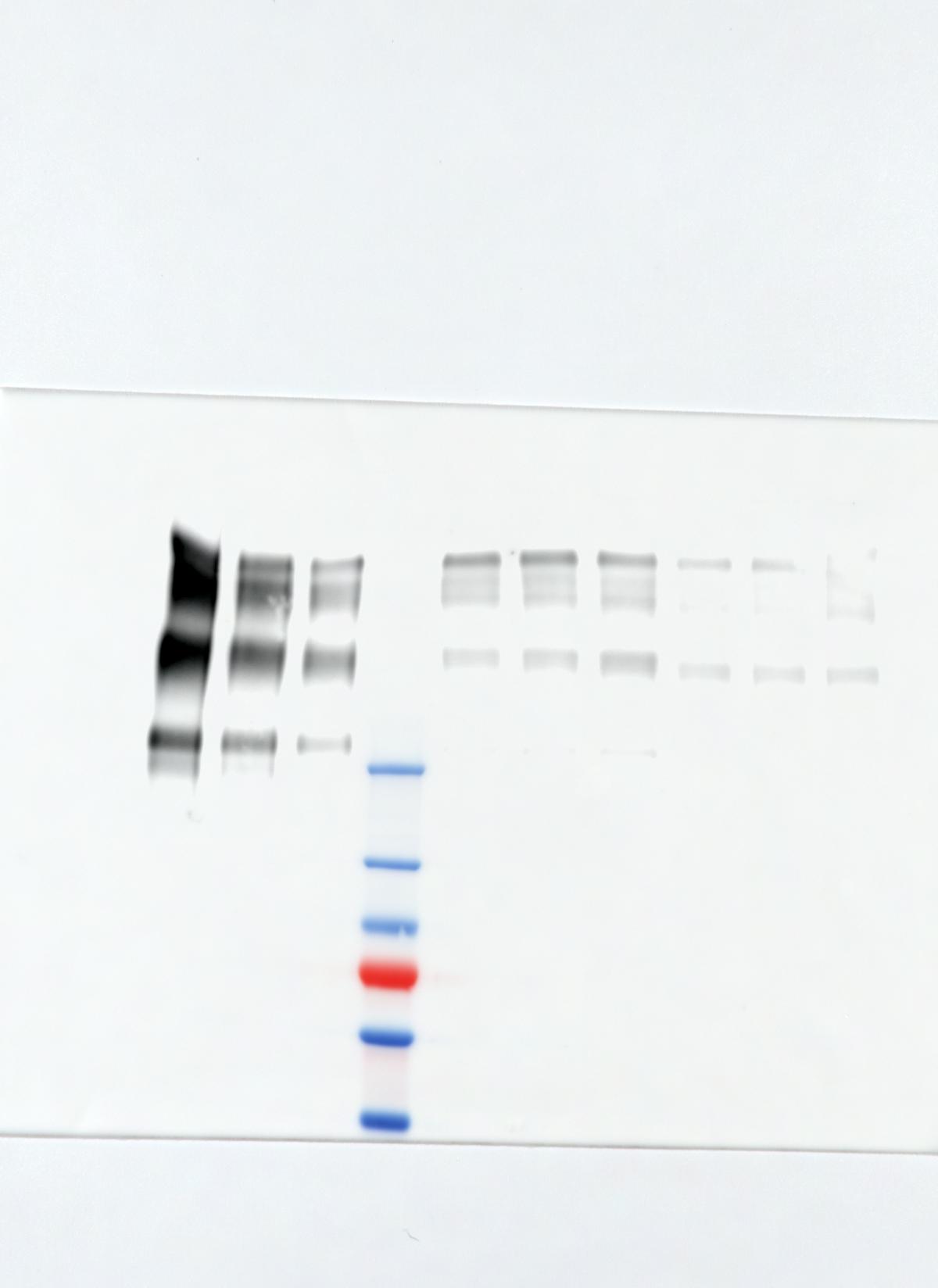

Supplement: Figure 5—figure supplement 2—source data 2. [file elife-104465-fig5-figsupp2-data2.zip › Figure 5-figure supplement 2-source data 2/Figure 5-figure supplement 2B Day5.jpg]

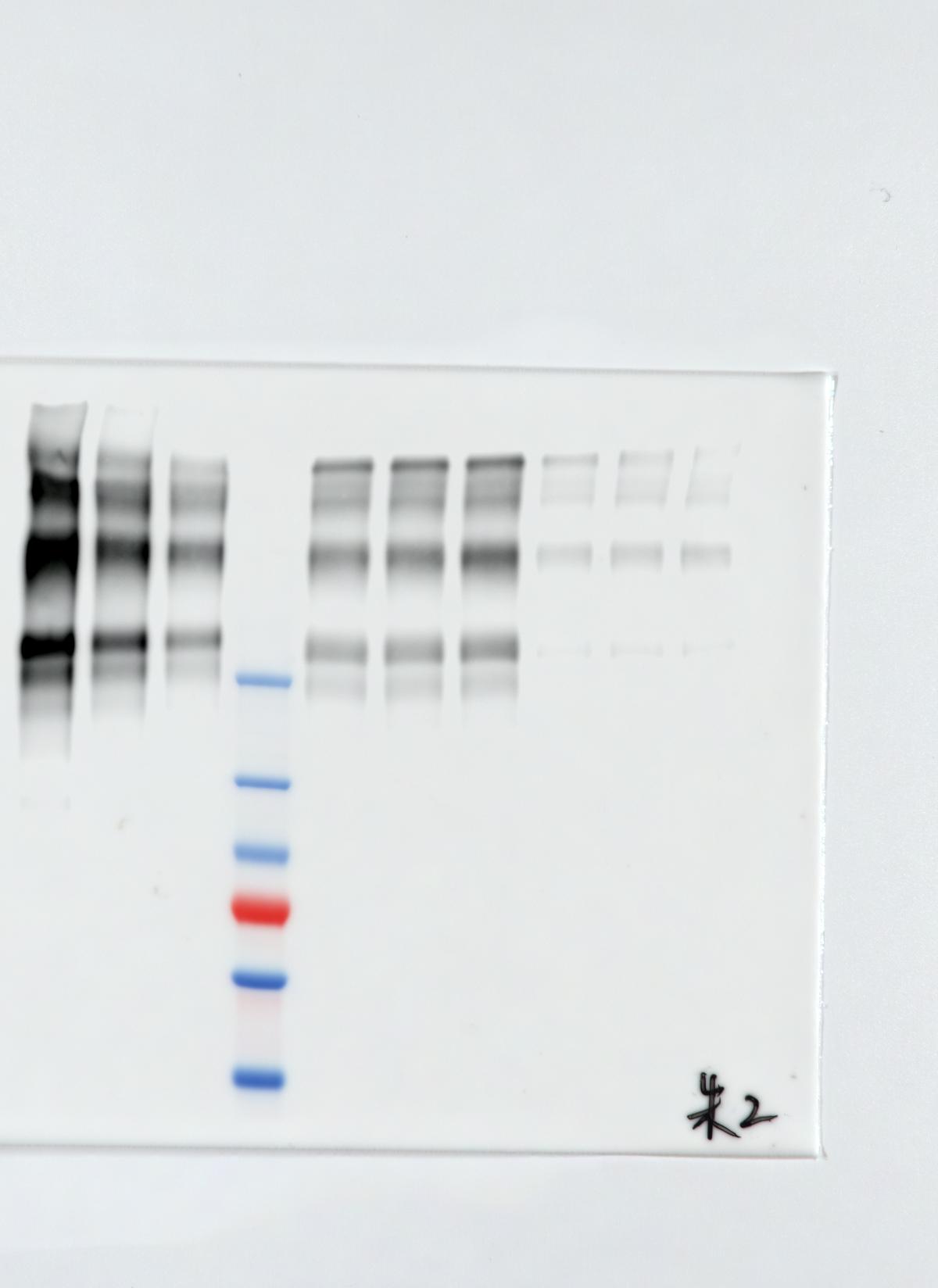

Supplement: Figure 5—figure supplement 2—source data 2. [file elife-104465-fig5-figsupp2-data2.zip › Figure 5-figure supplement 2-source data 2/Figure 5-figure supplement 2B Day7.jpg]

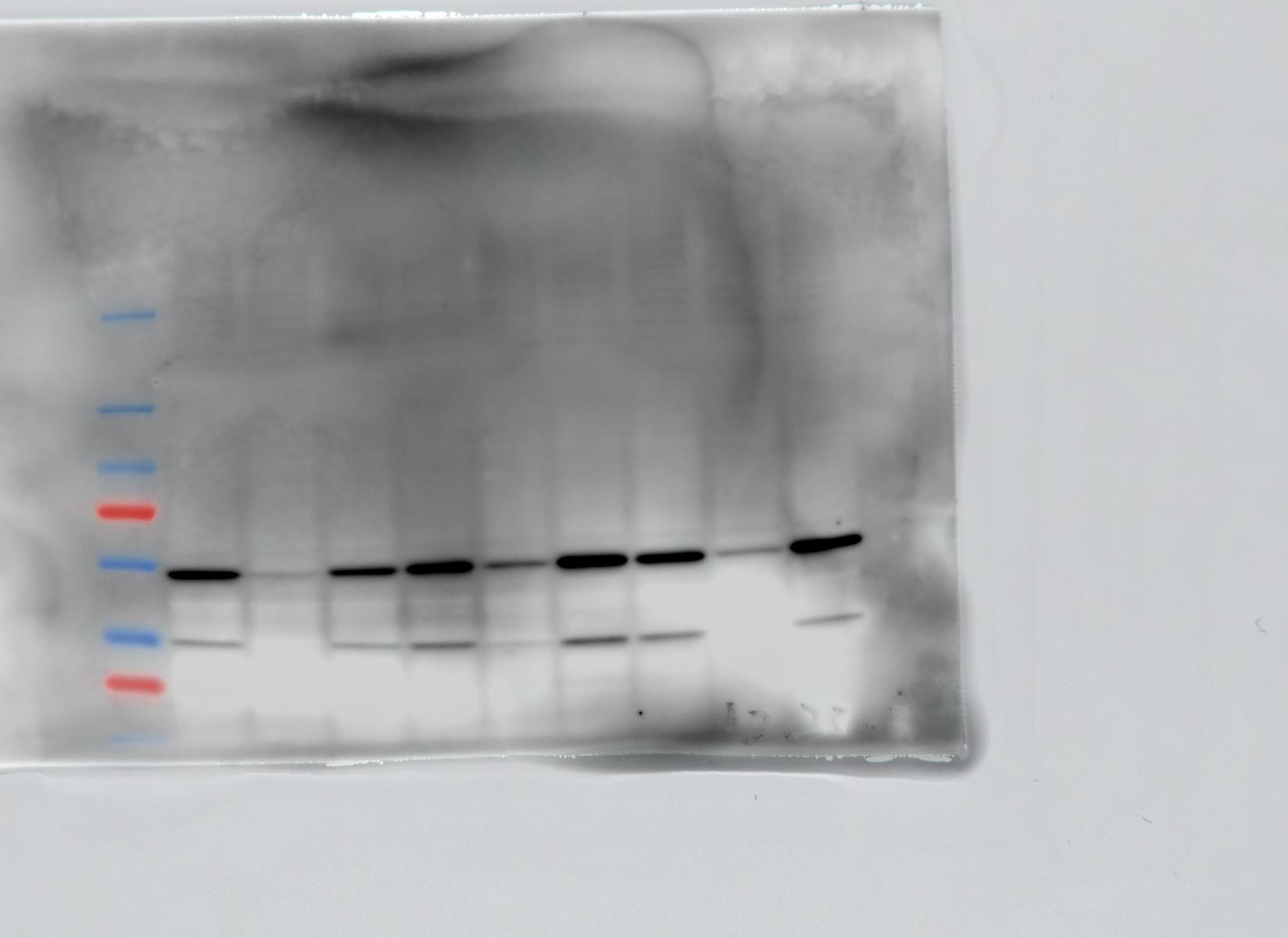

Supplement: Figure 6—source data 2. [file elife-104465-fig6-data2.zip › Figure 6-source data 2/Figure 6G LMBV-MCP.jpg]

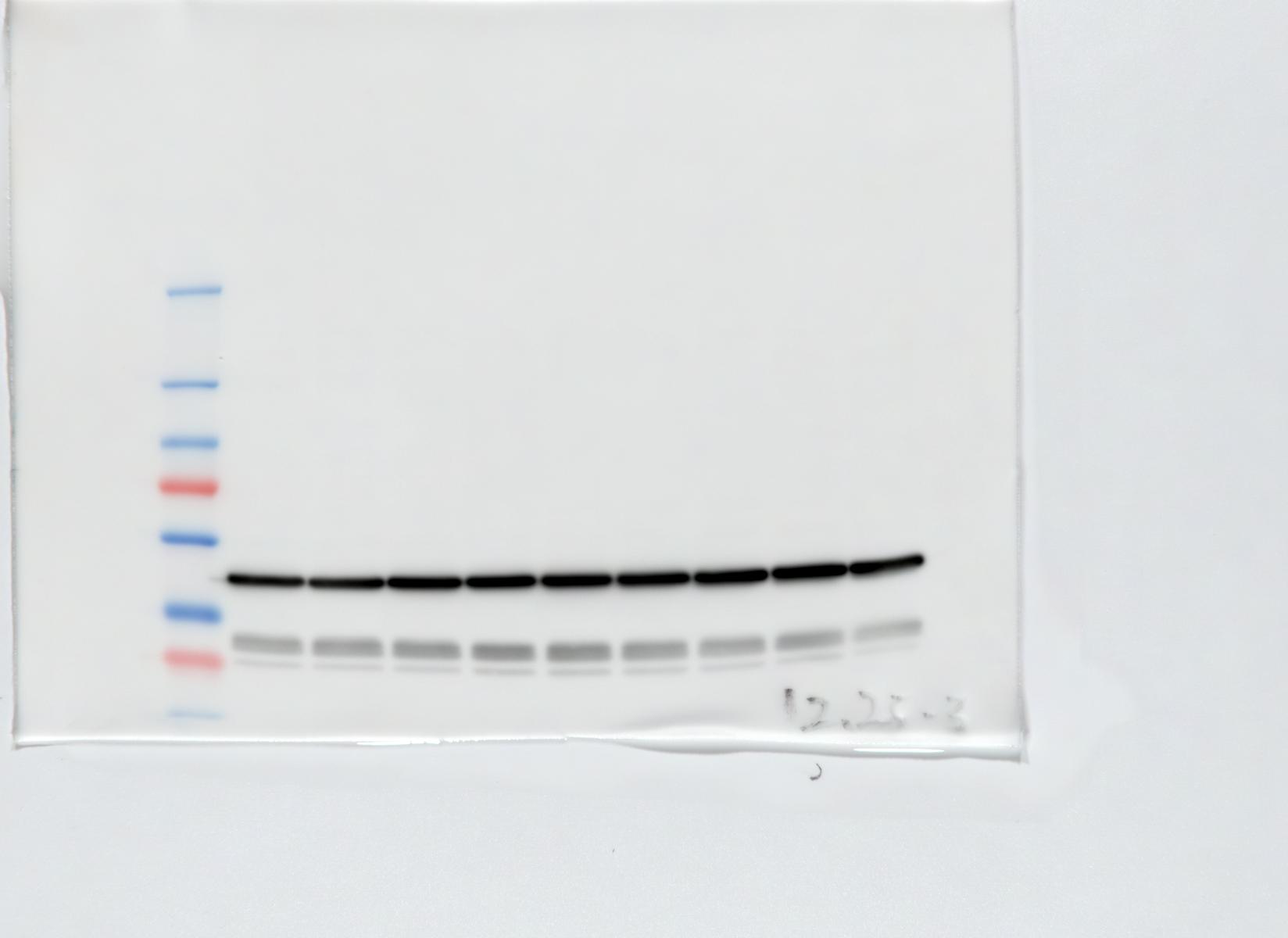

Supplement: Figure 6—source data 2. [file elife-104465-fig6-data2.zip › Figure 6-source data 2/Figure 6G β-actin.jpg]

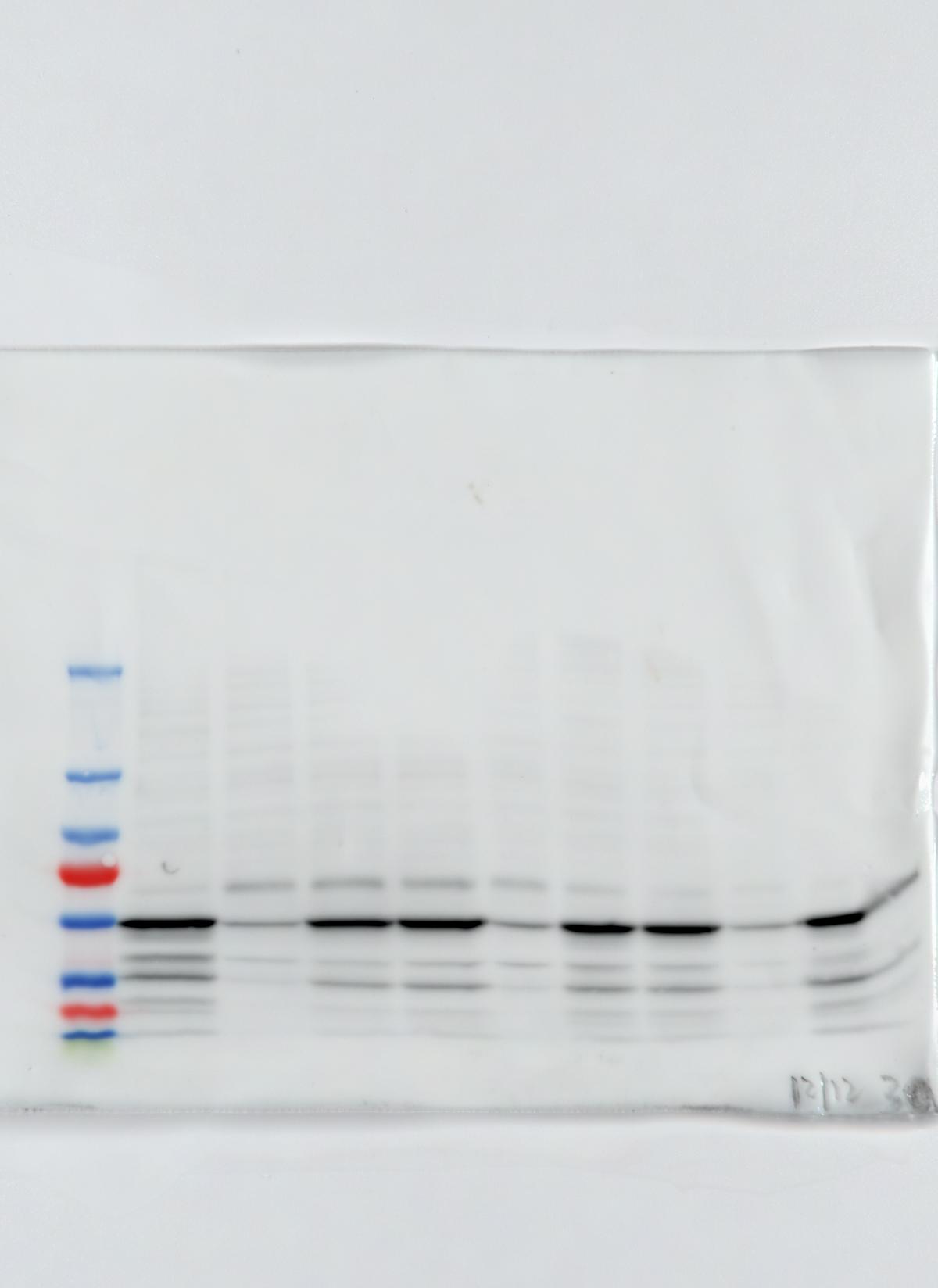

Supplement: Figure 6—source data 2. [file elife-104465-fig6-data2.zip › Figure 6-source data 2/Figure 6H LMBV-MCP.jpg]

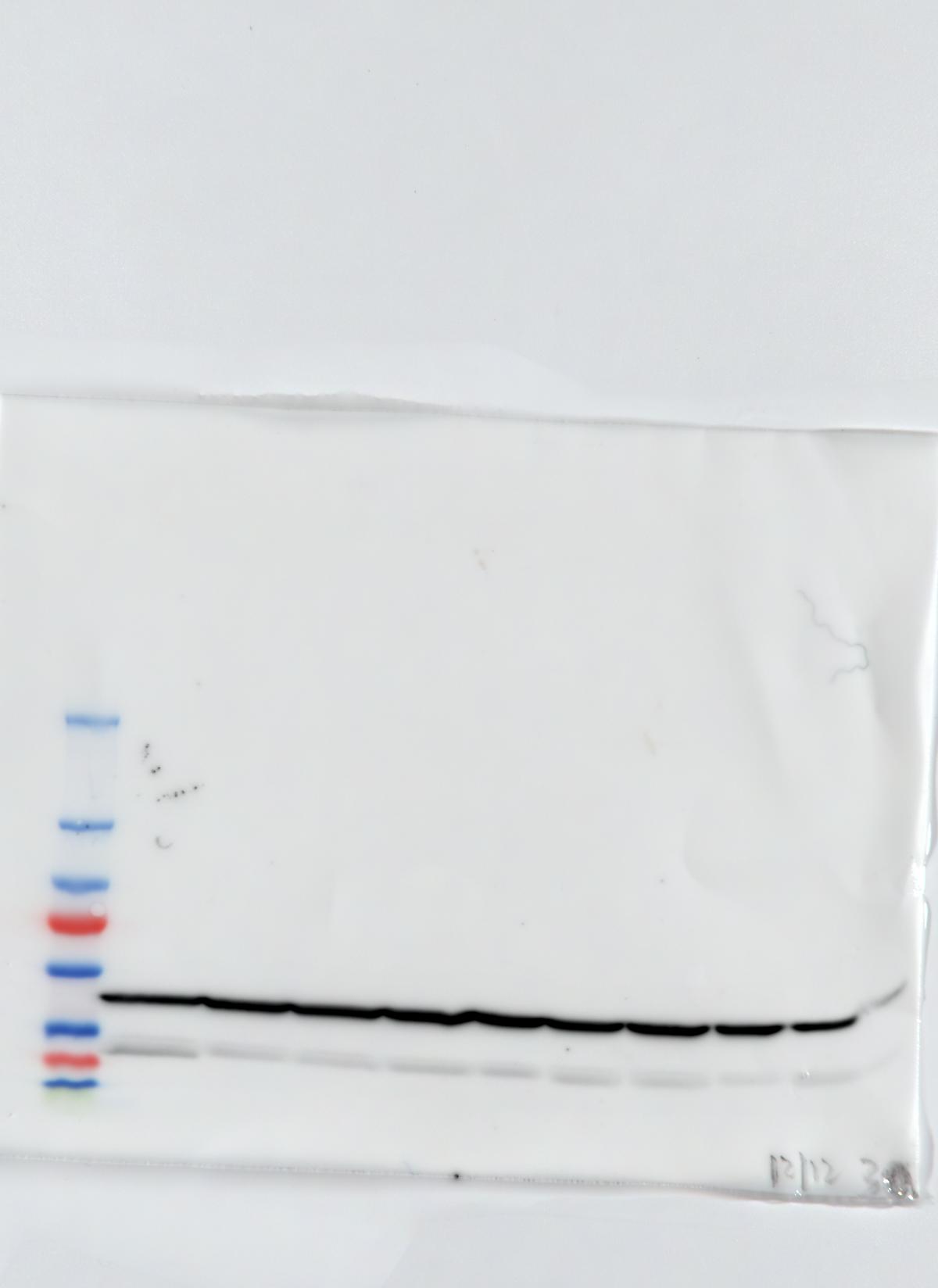

Supplement: Figure 6—source data 2. [file elife-104465-fig6-data2.zip › Figure 6-source data 2/Figure 6H β-actin.jpg]

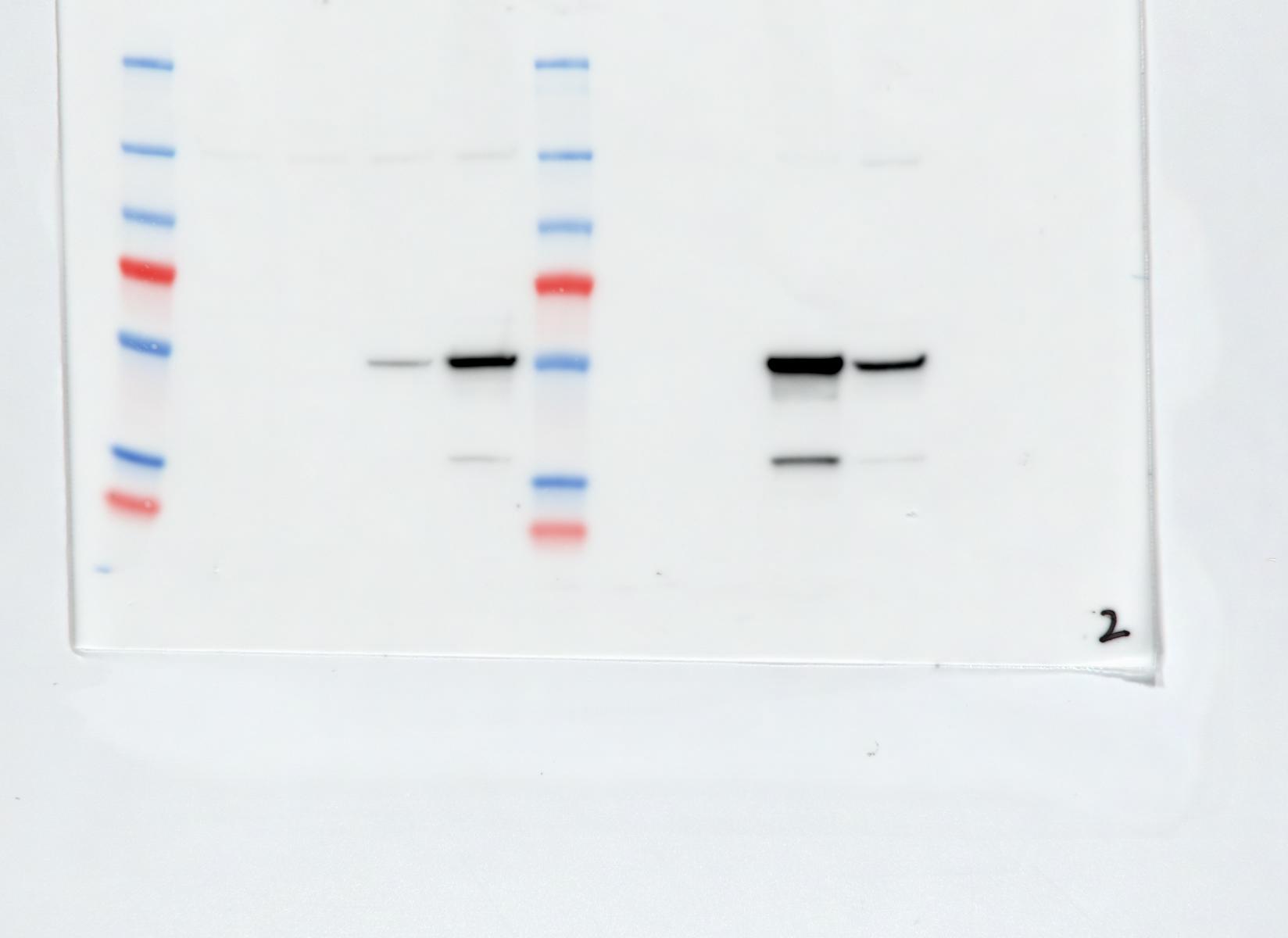

Supplement: Figure 6—figure supplement 2—source data 2. [file elife-104465-fig6-figsupp2-data2.zip › Figure 6-figure supplement 2-source data 2/Figure 6-figure supplement 2C LMBV-MCP.jpg]

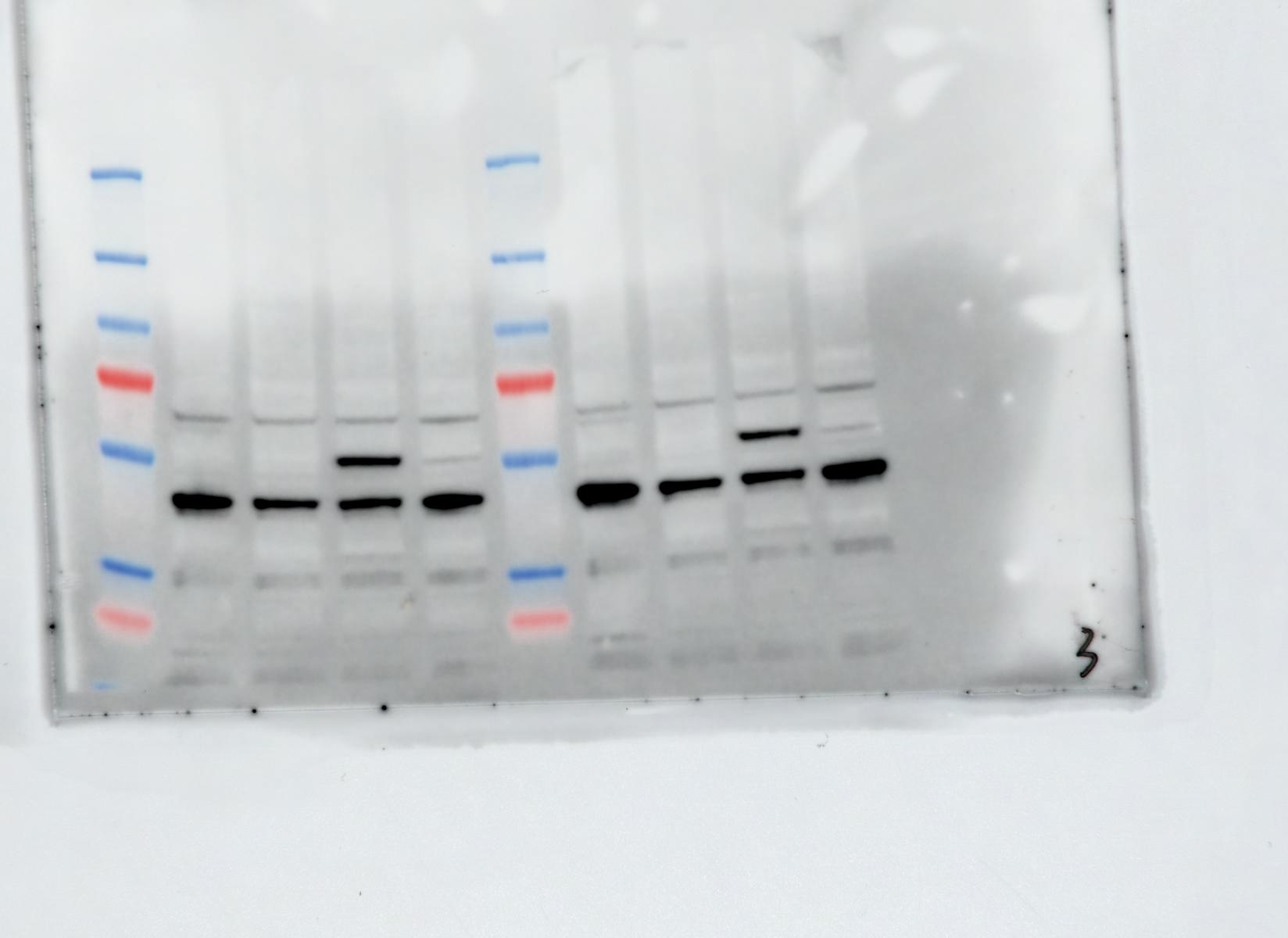

Supplement: Figure 6—figure supplement 2—source data 2. [file elife-104465-fig6-figsupp2-data2.zip › Figure 6-figure supplement 2-source data 2/Figure 6-figure supplement 2C β-actin.jpg]

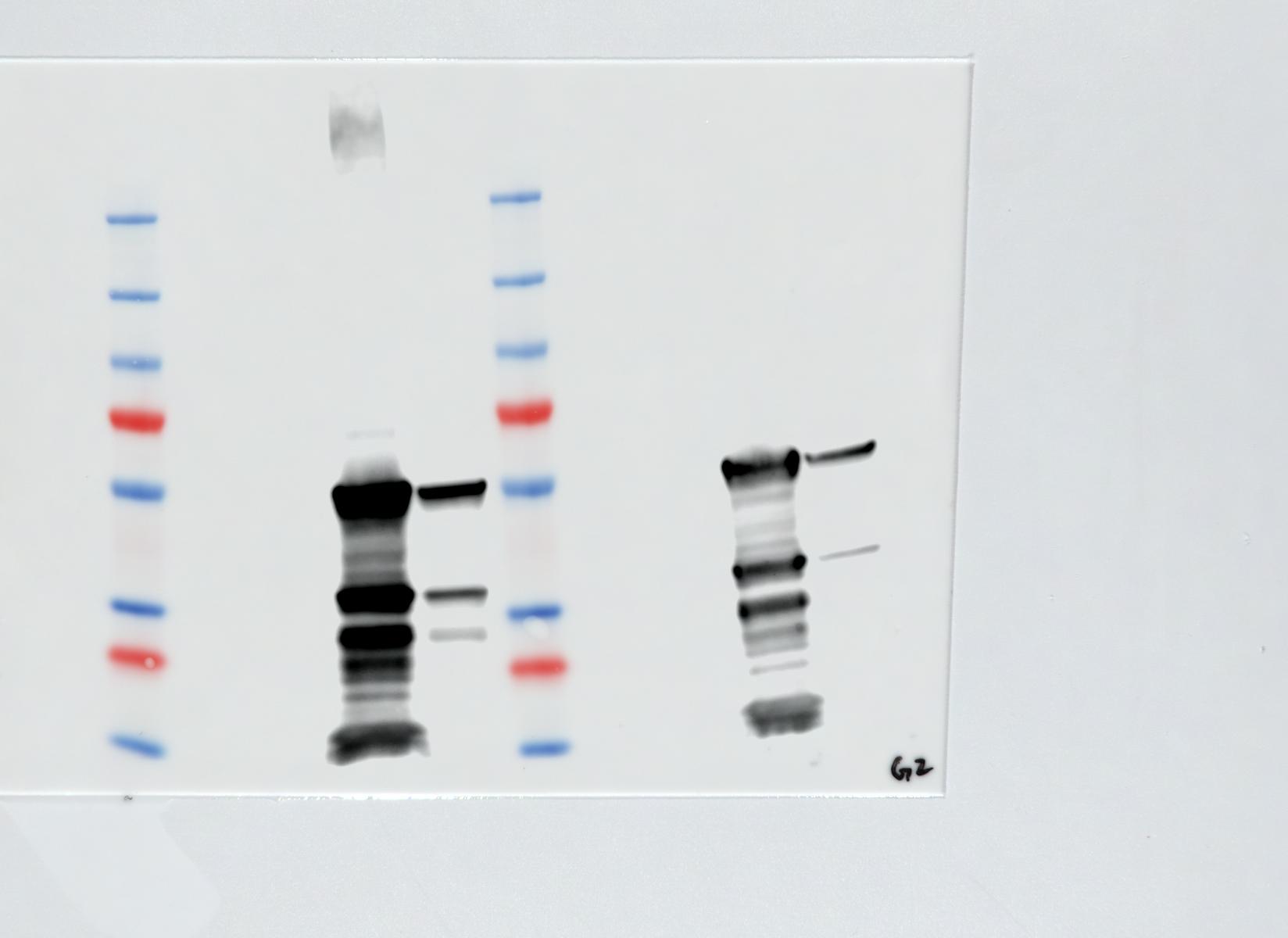

Supplement: Figure 6—figure supplement 2—source data 2. [file elife-104465-fig6-figsupp2-data2.zip › Figure 6-figure supplement 2-source data 2/Figure 6-figure supplement 2E LMBV-MCP.jpg]

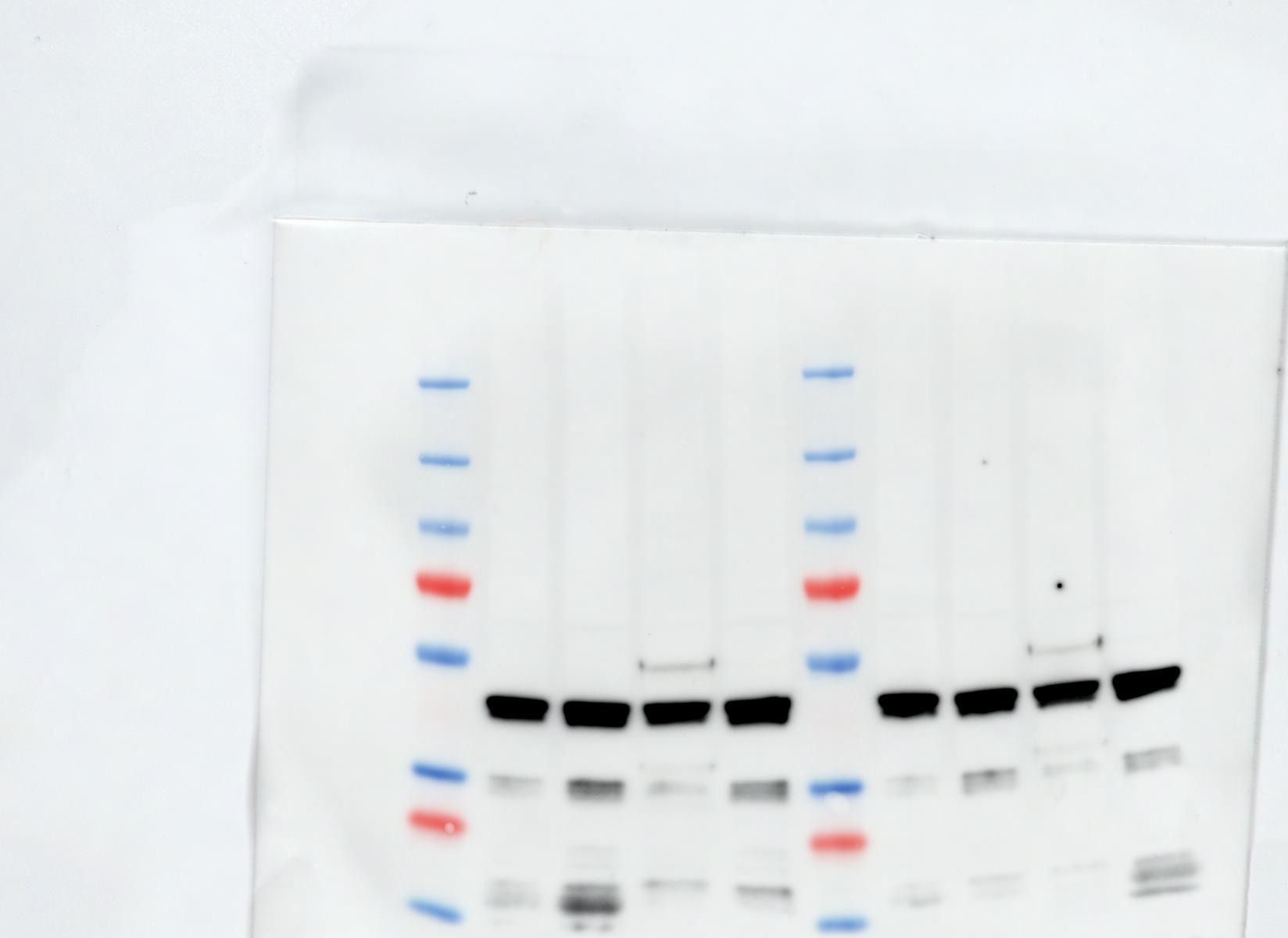

Supplement: Figure 6—figure supplement 2—source data 2. [file elife-104465-fig6-figsupp2-data2.zip › Figure 6-figure supplement 2-source data 2/Figure 6-figure supplement 2E β-actin.jpg]

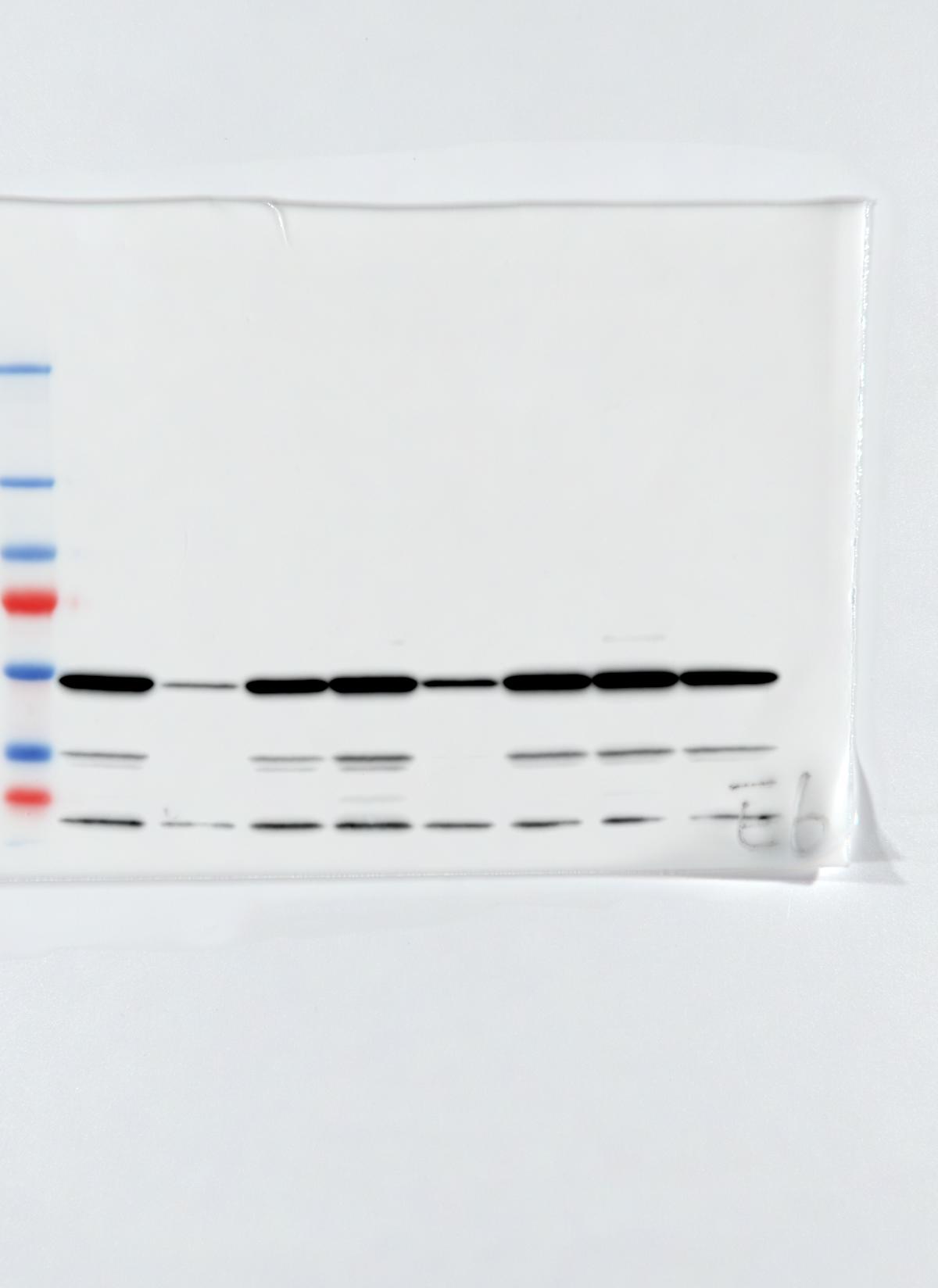

Supplement: Figure 7—source data 2. [file elife-104465-fig7-data2.zip › Figure 7-source data 2/Figure 7D 42DPI-S LMBV-MCP.jpg]

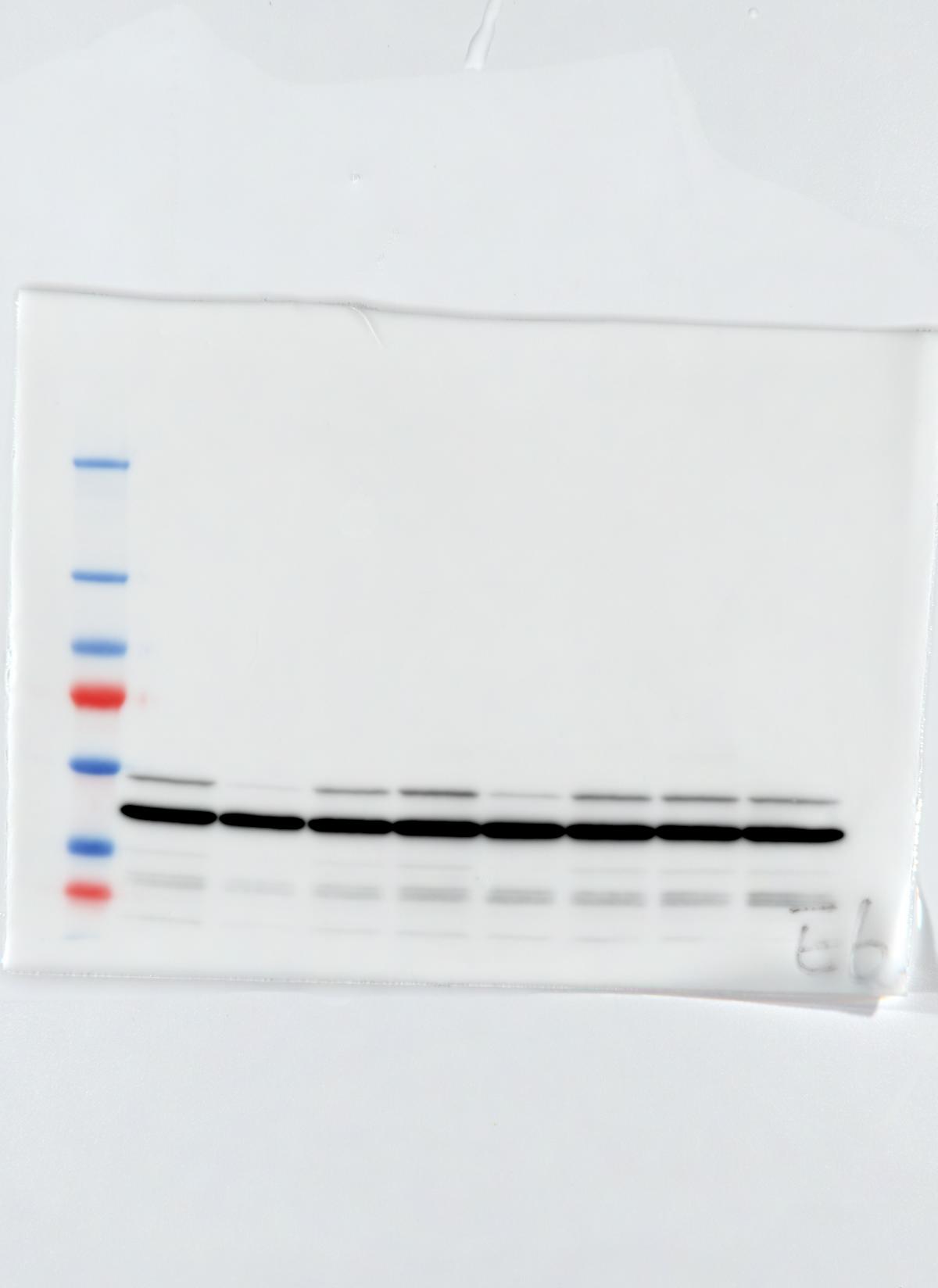

Supplement: Figure 7—source data 2. [file elife-104465-fig7-data2.zip › Figure 7-source data 2/Figure 7D 42DPI-S β-actin.jpg]

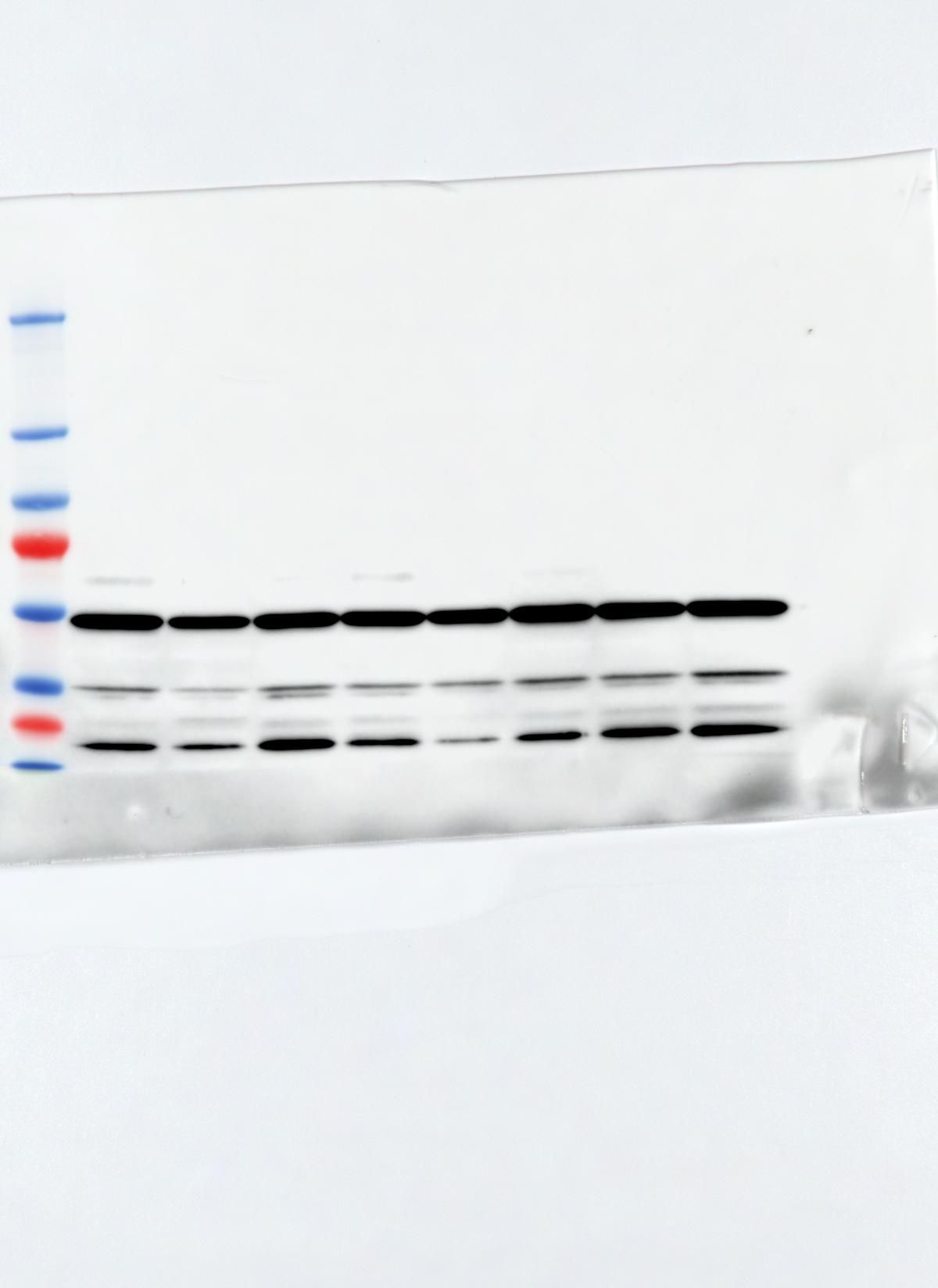

Supplement: Figure 7—source data 2. [file elife-104465-fig7-data2.zip › Figure 7-source data 2/Figure 7D 42DPI-S-IgMDEP LMBV-MCP.jpg]

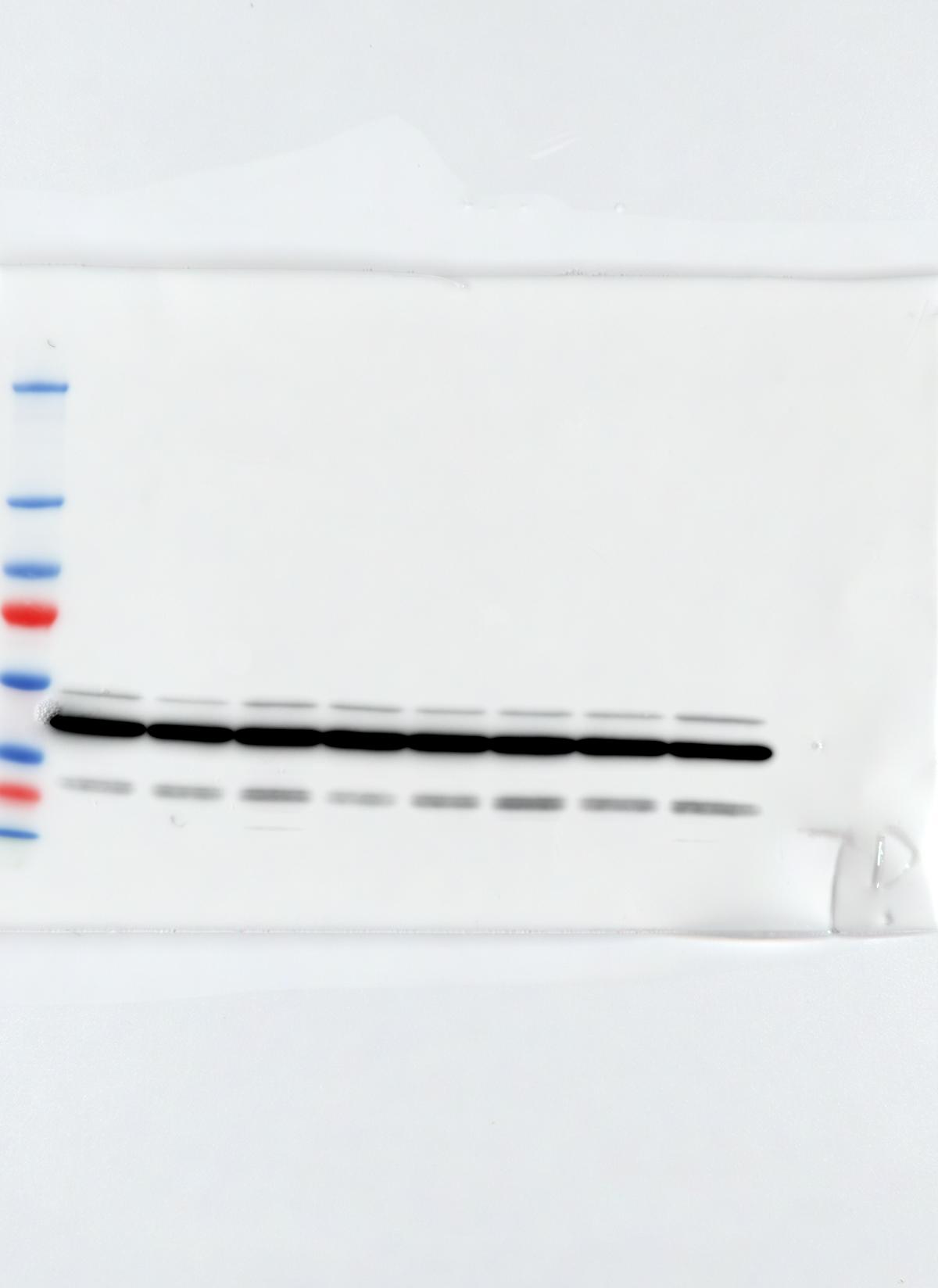

Supplement: Figure 7—source data 2. [file elife-104465-fig7-data2.zip › Figure 7-source data 2/Figure 7D 42DPI-S-IgMDEP β-actin.jpg]

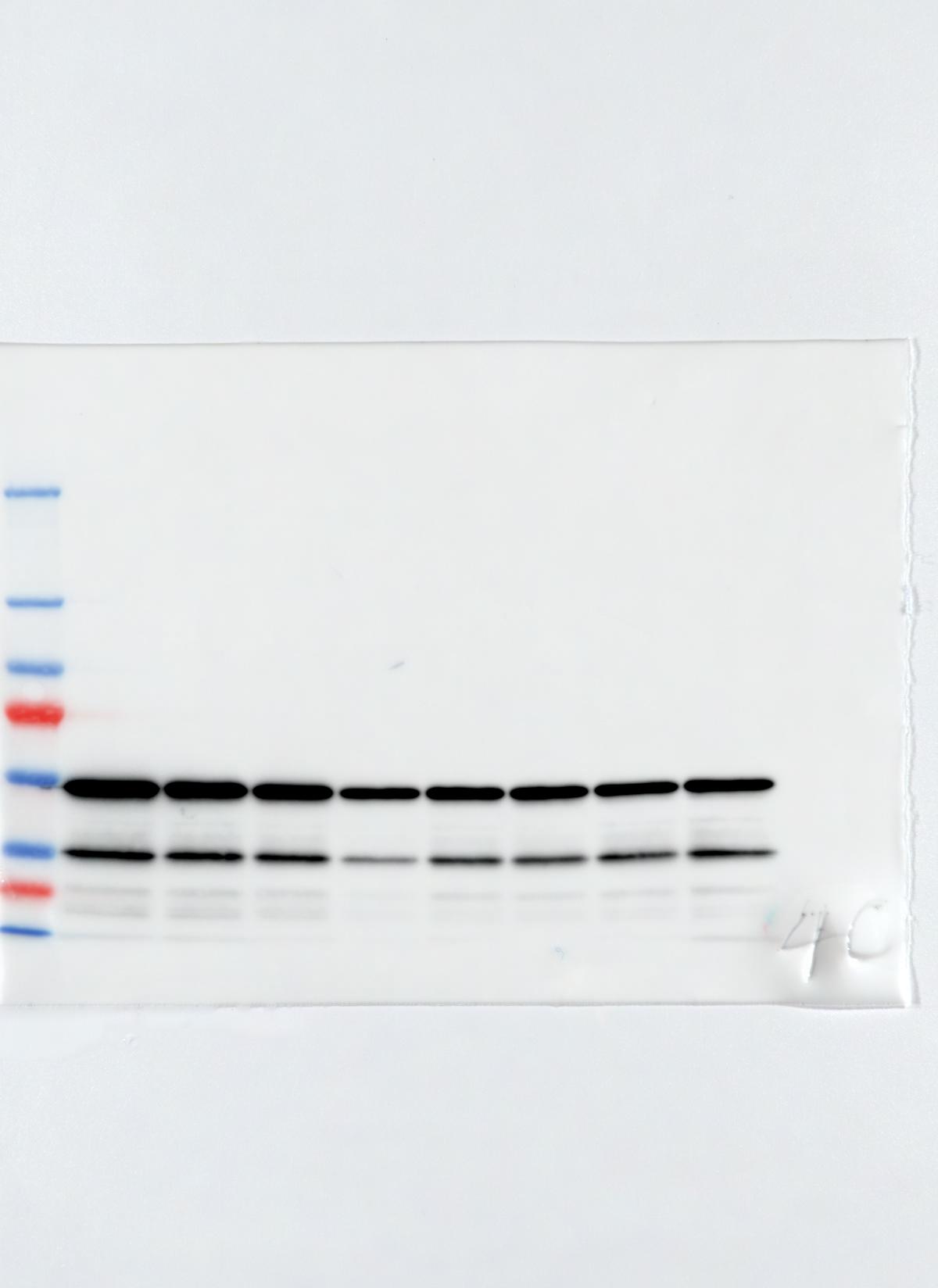

Supplement: Figure 7—source data 2. [file elife-104465-fig7-data2.zip › Figure 7-source data 2/Figure 7D Control LMBV-MCP.jpg]

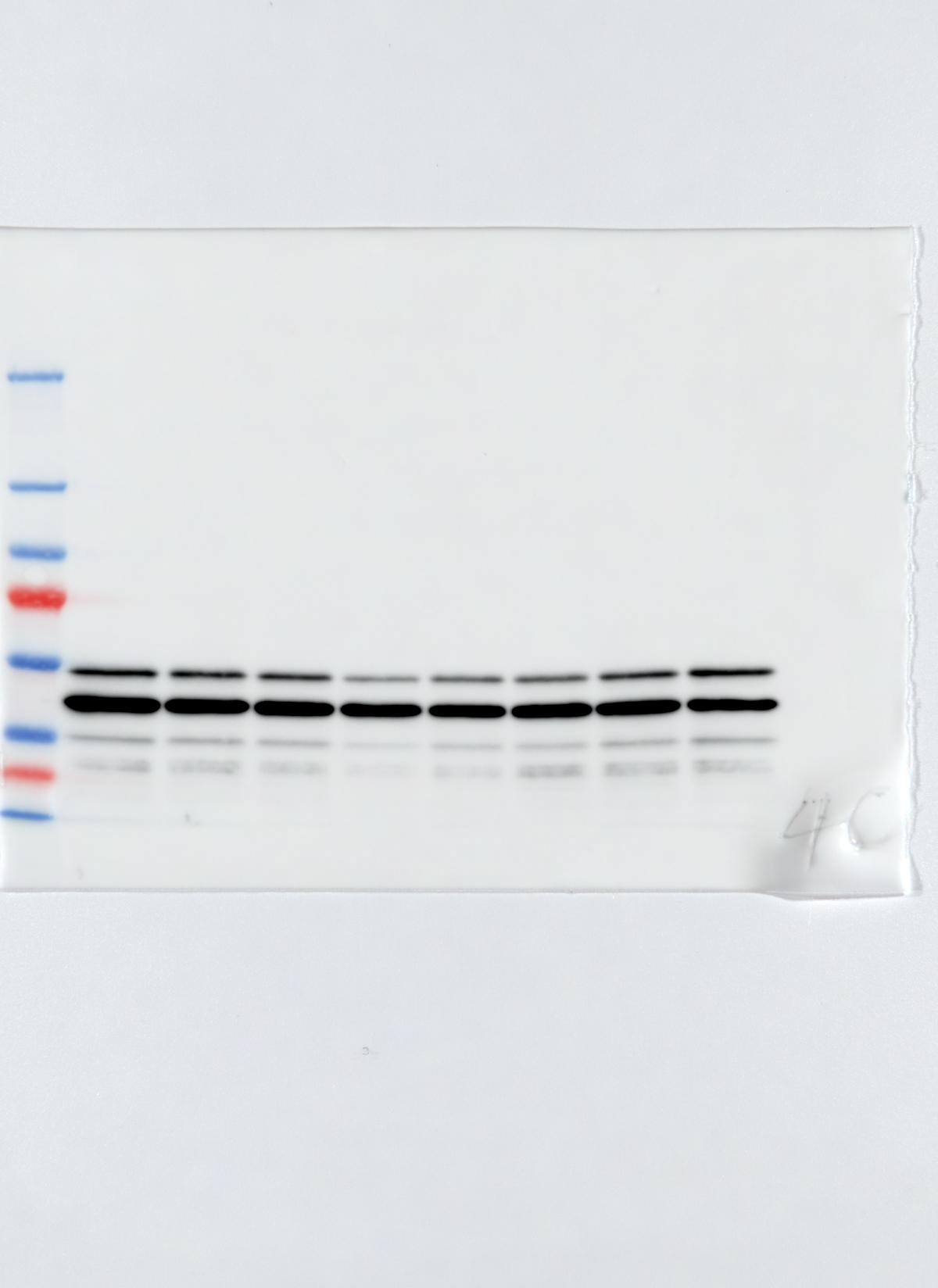

Supplement: Figure 7—source data 2. [file elife-104465-fig7-data2.zip › Figure 7-source data 2/Figure 7D Control β-actin.jpg]

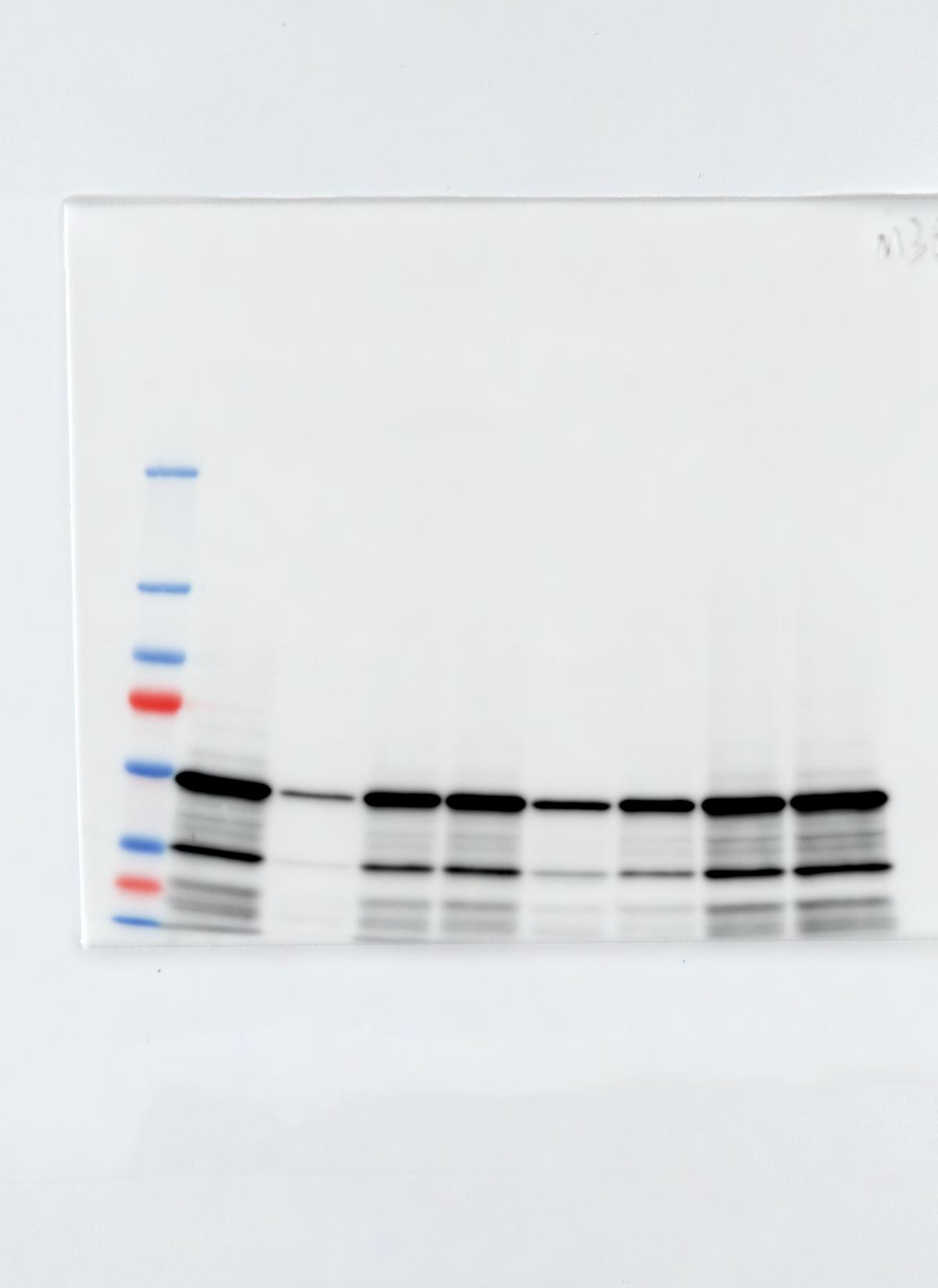

Supplement: Figure 7—source data 2. [file elife-104465-fig7-data2.zip › Figure 7-source data 2/Figure 7F 42DPI-S LMBV-MCP.jpg]

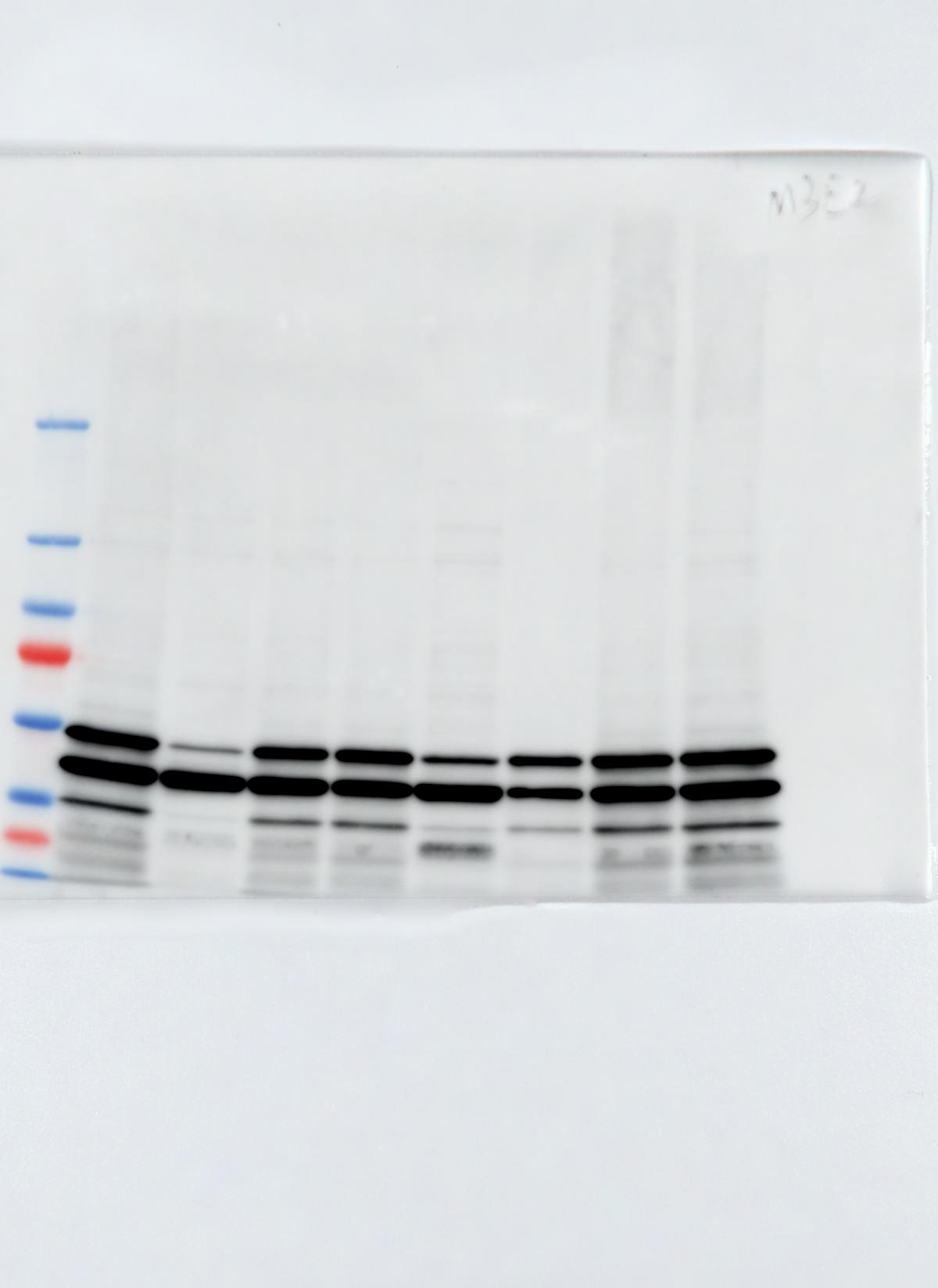

Supplement: Figure 7—source data 2. [file elife-104465-fig7-data2.zip › Figure 7-source data 2/Figure 7F 42DPI-S β-actin.jpg]

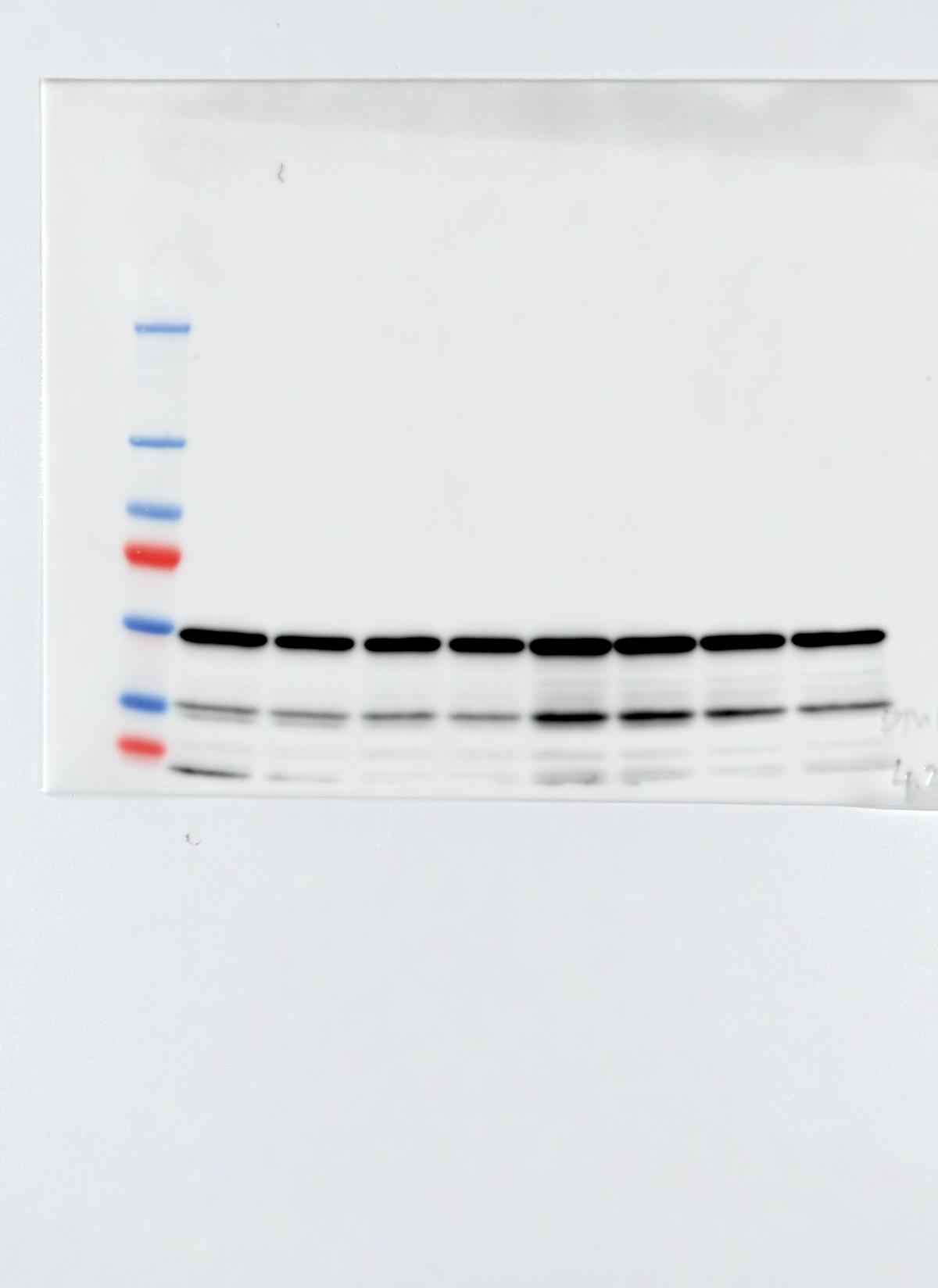

Supplement: Figure 7—source data 2. [file elife-104465-fig7-data2.zip › Figure 7-source data 2/Figure 7F 42DPI-S-IgMDEP LMBV-MCP.jpg]

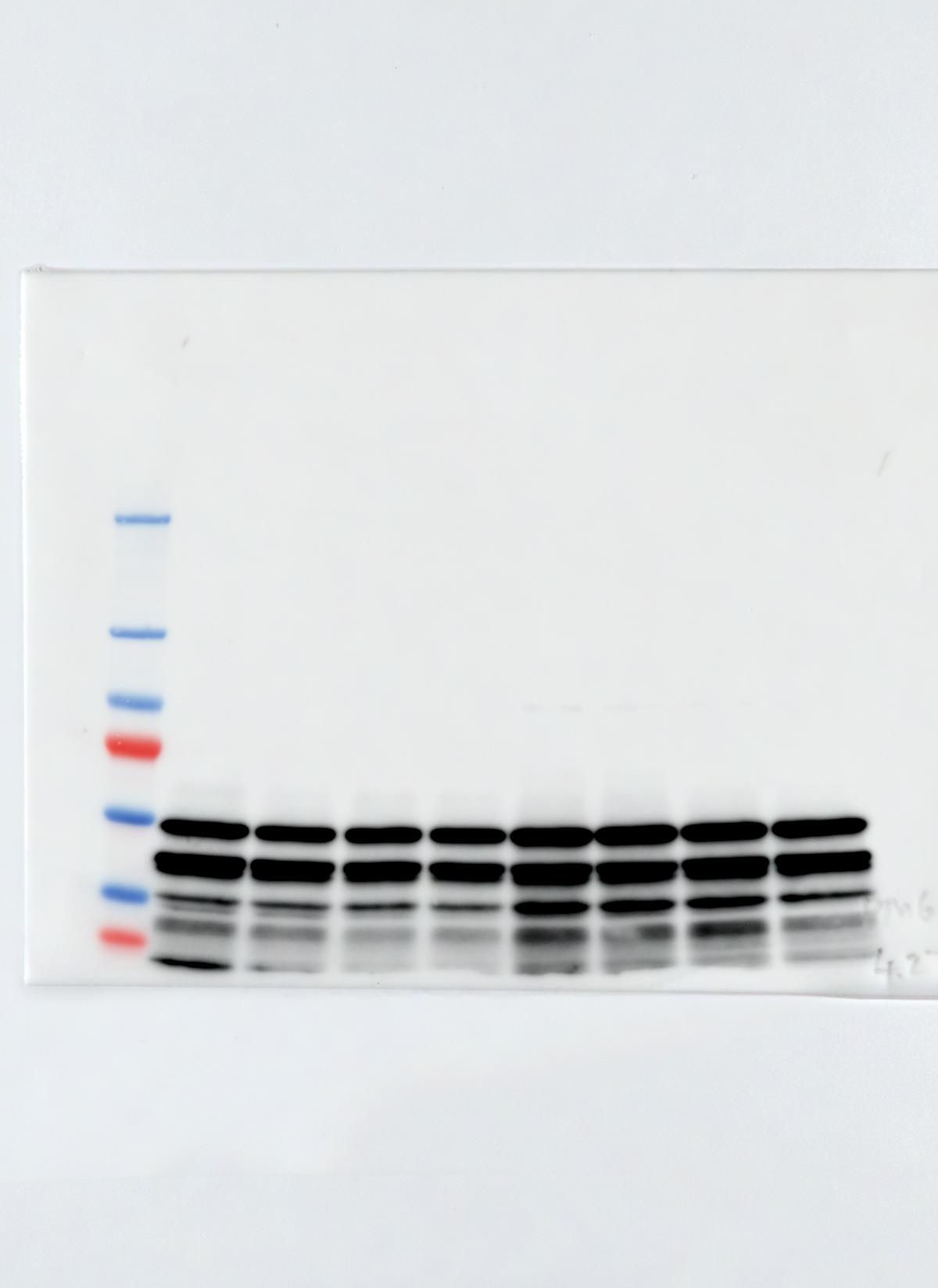

Supplement: Figure 7—source data 2. [file elife-104465-fig7-data2.zip › Figure 7-source data 2/Figure 7F 42DPI-S-IgMDEP β-actin.jpg]

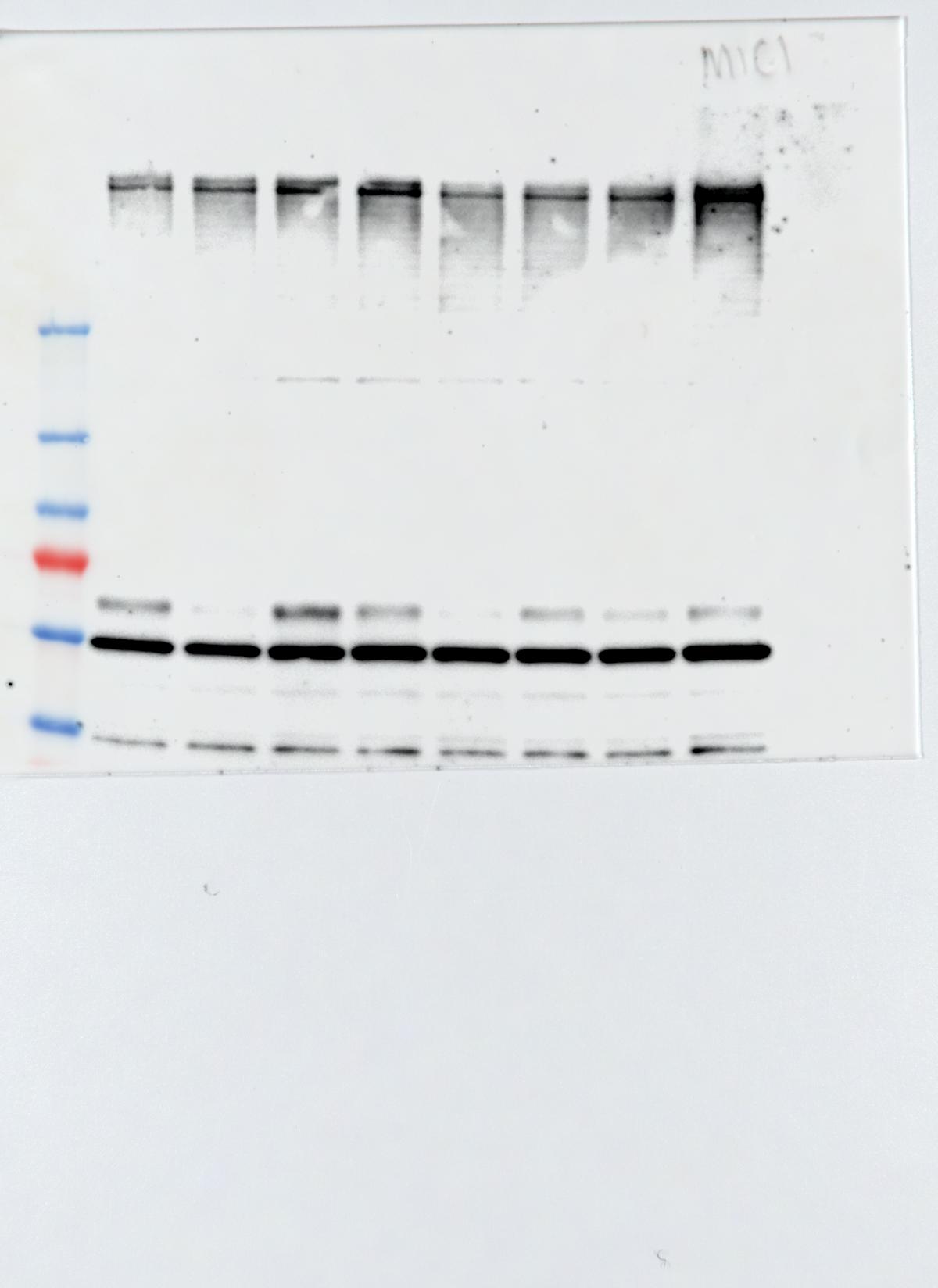

Supplement: Figure 7—source data 2. [file elife-104465-fig7-data2.zip › Figure 7-source data 2/Figure 7F Control LMBV-MCP.jpg]

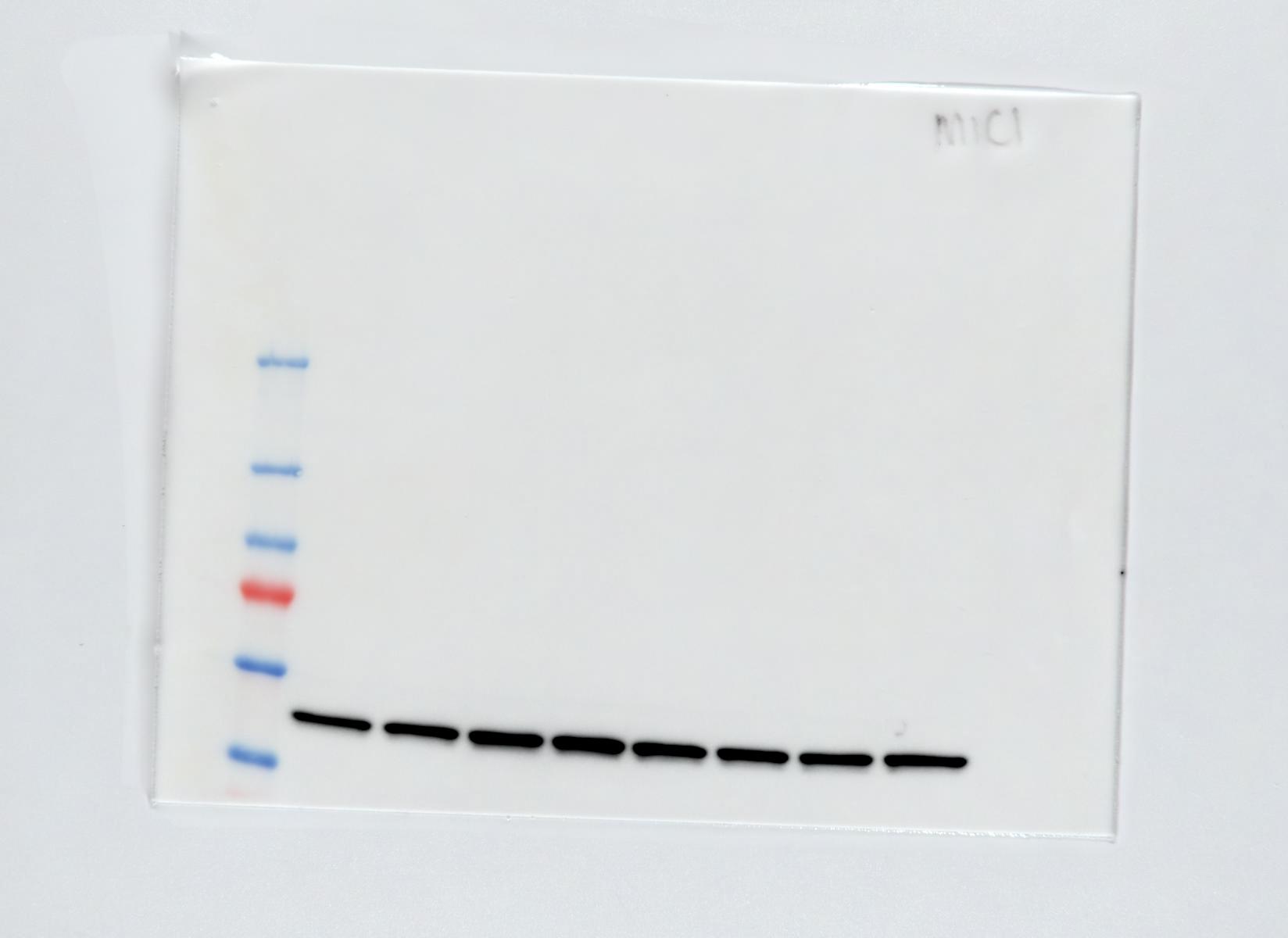

Supplement: Figure 7—source data 2. [file elife-104465-fig7-data2.zip › Figure 7-source data 2/Figure 7F Control β-actin.jpg]
