# Supplementary material for: Newly discovered and conserved role of IgM against viral infection in an early vertebrate
Source: eLife. 2025 Sep 4;14:RP104465. doi: 10.7554/eLife.104465 (PMC12410970; doi:10.7554/eLife.104465)
Supplement: Figure 3—figure supplement 2—source data 1. [file elife-104465-fig3-figsupp2-data1.pdf]

Figure 3–figure supplement 2

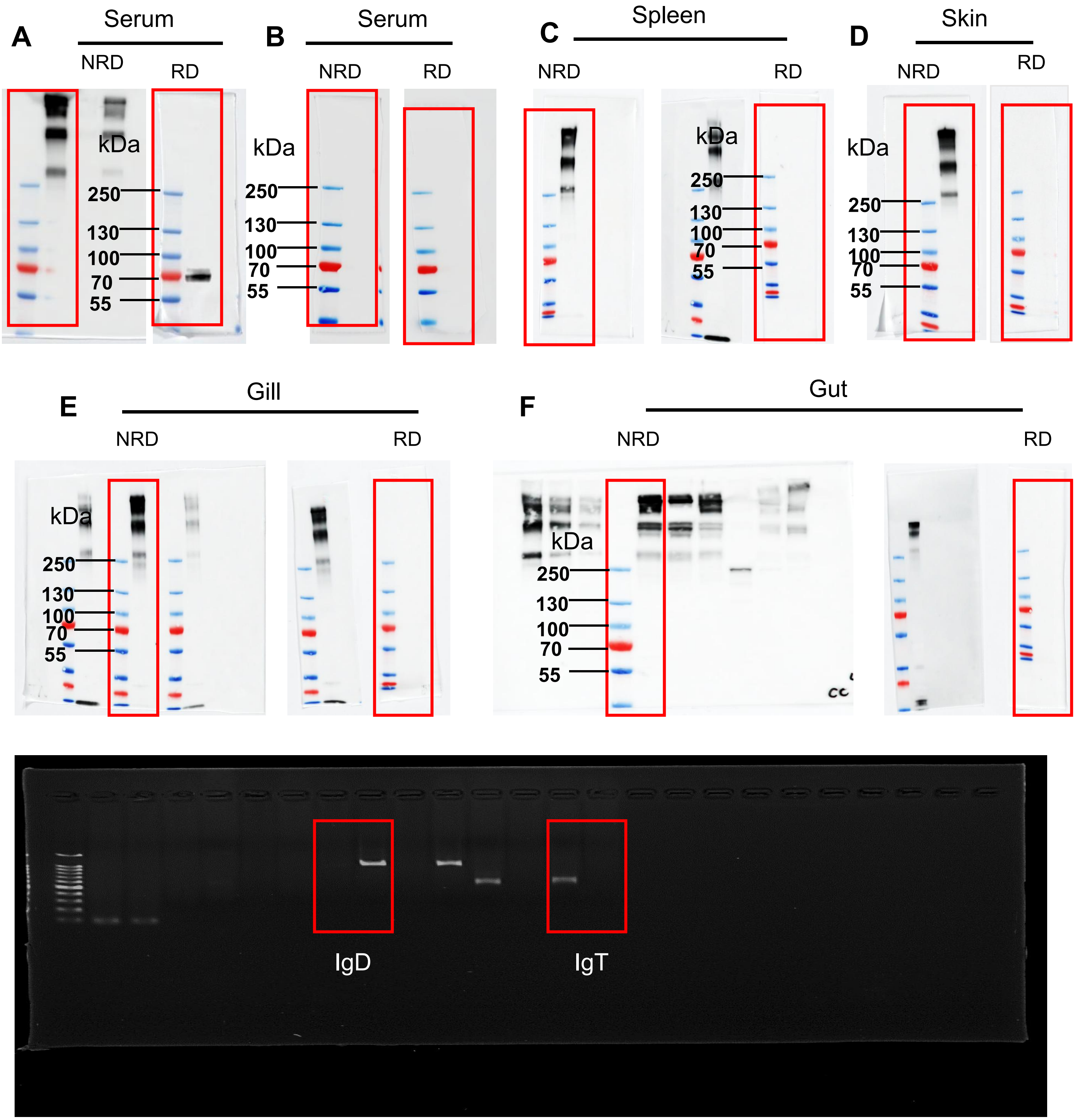

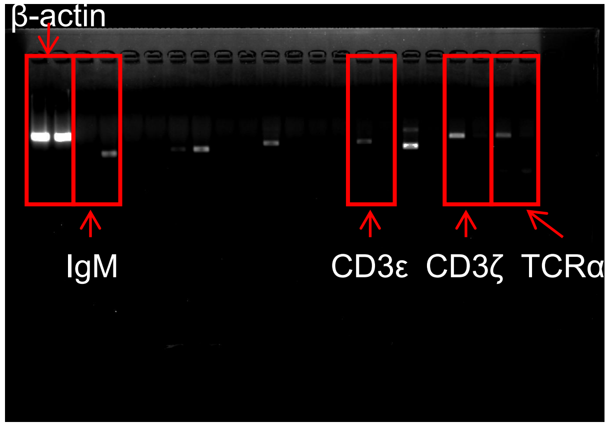

Figure 3–figure supplement 2 Original membranes corresponding to Figure 3–figure supplement 2A, 2B,2C, 2D, 2E, 2F, and 2H. Rainbow molecular weight markers were employed. The areas highlighted by red boxes are used in this result figure.
