# Supplementary material for: Newly discovered and conserved role of IgM against viral infection in an early vertebrate
Source: eLife. 2025 Sep 4;14:RP104465. doi: 10.7554/eLife.104465 (PMC12410970; doi:10.7554/eLife.104465)
Supplement: Figure 5—figure supplement 2—source data 1. [file elife-104465-fig5-figsupp2-data1.pdf]

Figure 5–figure supplement 2A

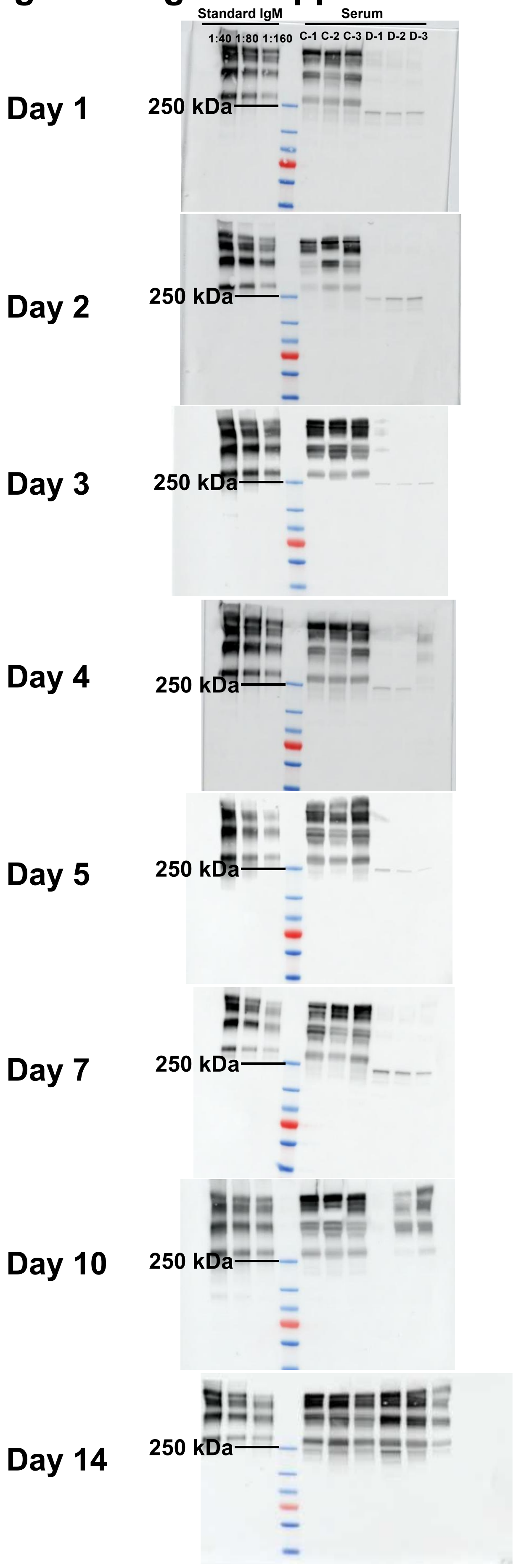

Figure 5–figure supplement 2B

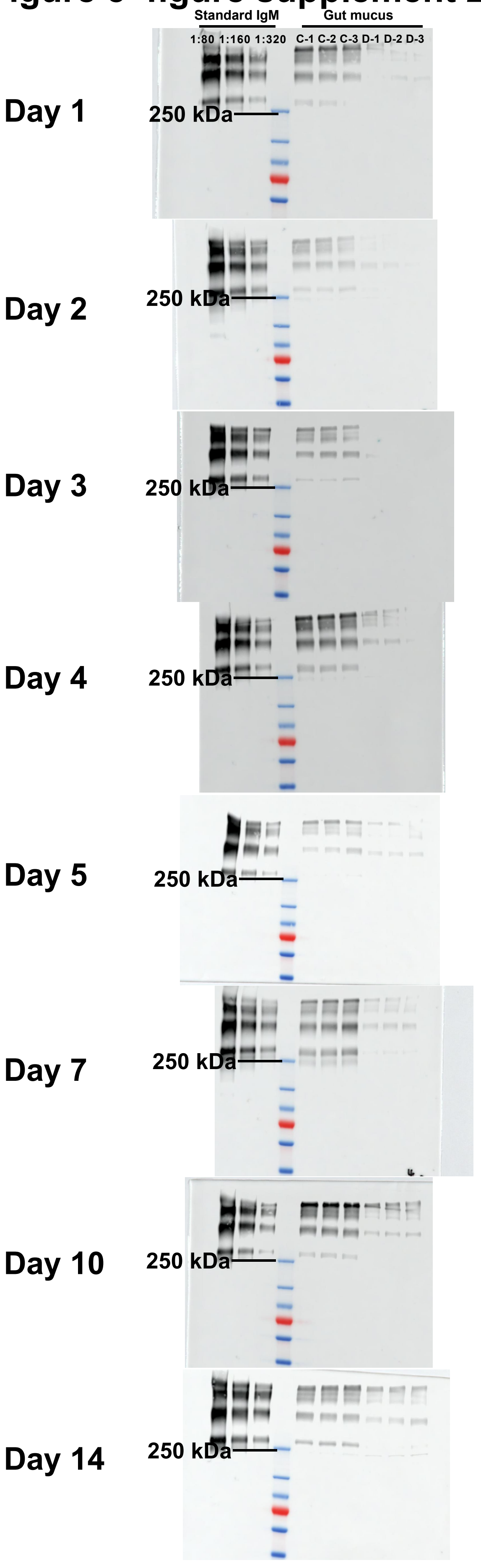

Figure 5–figure supplement 2 Original membranes corresponding to Figure 5–figure supplement 2A and 2B. The left membranes correspond to serum IgM, and the right membranes correspond to gut mucus IgM. Rainbow molecular weight markers were employed. The areas highlighted by red boxes are used in this result figure.
