# Supplementary material for: Newly discovered and conserved role of IgM against viral infection in an early vertebrate
Source: eLife. 2025 Sep 4;14:RP104465. doi: 10.7554/eLife.104465 (PMC12410970; doi:10.7554/eLife.104465)
Supplement: Figure 6—figure supplement 2—source data 1. [file elife-104465-fig6-figsupp2-data1.pdf]

Figure 6–figure supplement 2C

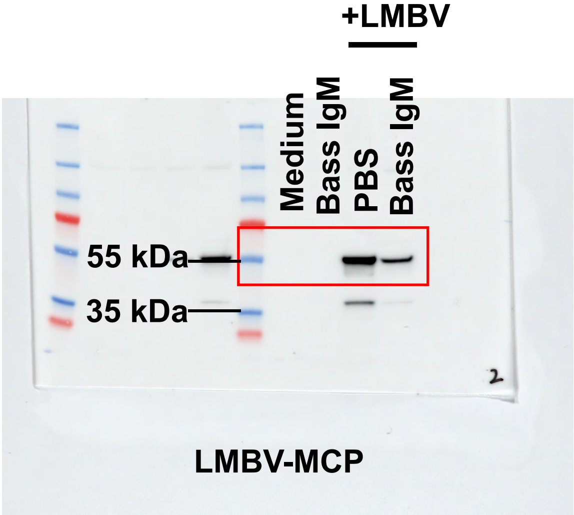

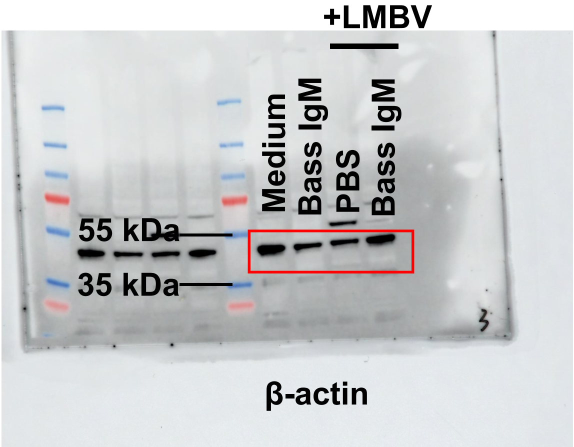

Figure 6–figure supplement 2E

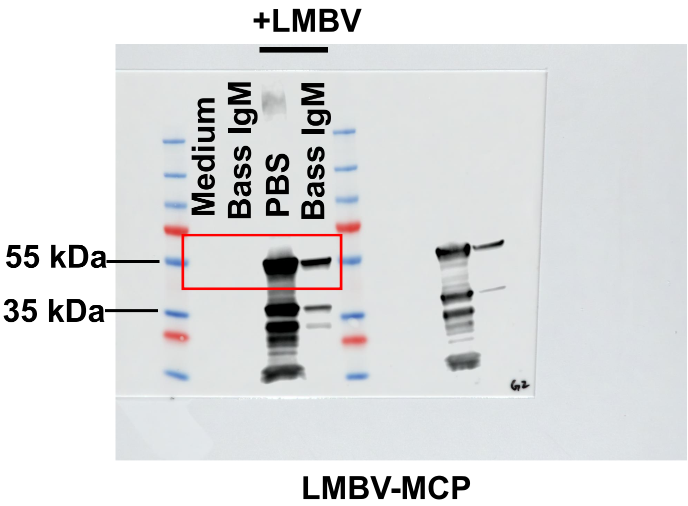

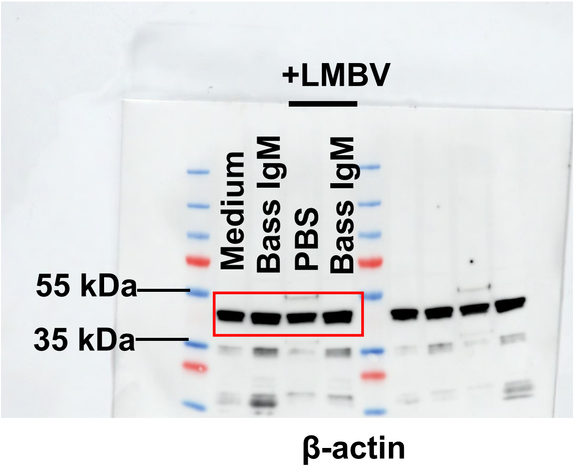

Figure 6–figure supplement 2 Original membranes corresponding to Figure 6–figure supplement 2C and 2E. The left membranes correspond to LMBV-MCP, and the right membranes correspond to β-actin. Rainbow molecular weight markers were employed. The areas highlighted by red boxes are used in this result figure.
