# Supplementary material for: Newly discovered and conserved role of IgM against viral infection in an early vertebrate
Source: eLife. 2025 Sep 4;14:RP104465. doi: 10.7554/eLife.104465 (PMC12410970; doi:10.7554/eLife.104465)
Supplement: Figure 7—source data 1. [file elife-104465-fig7-data1.pdf]

Figure 7D

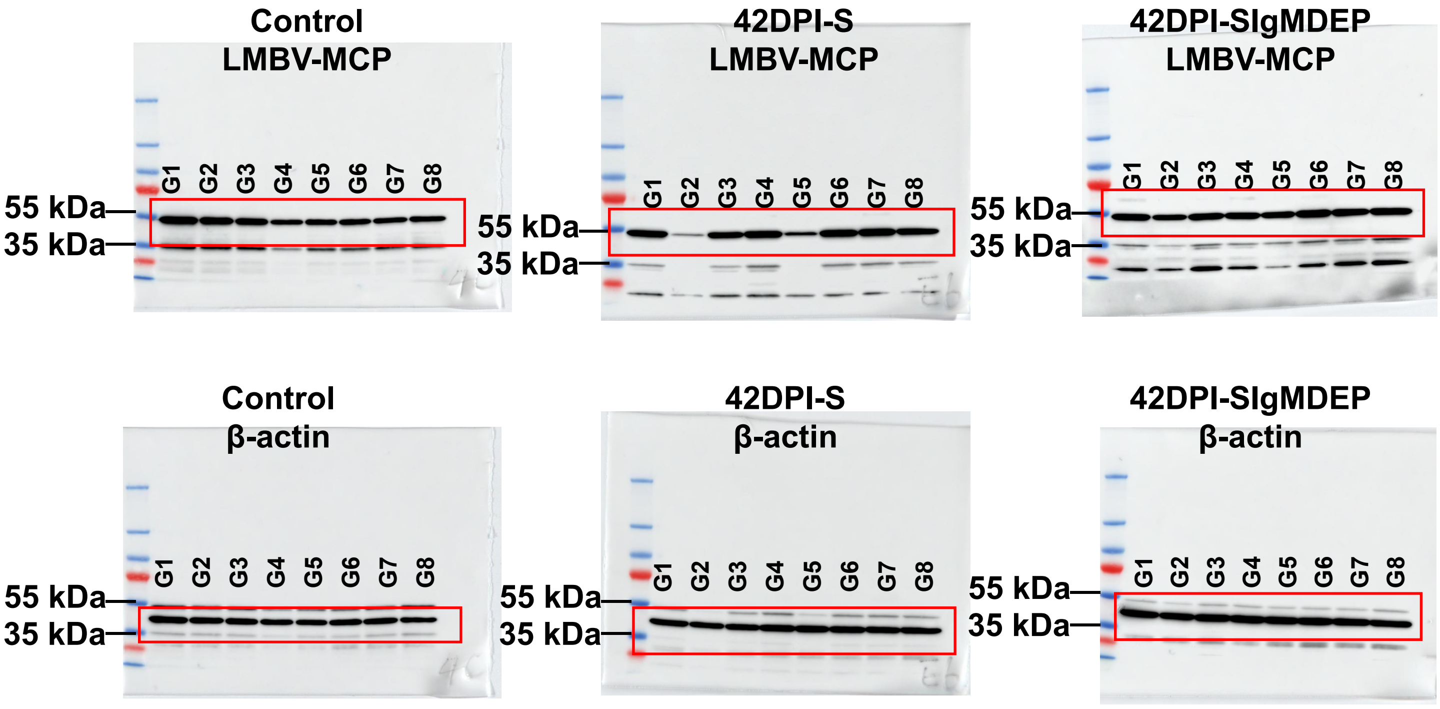

Figure 7F

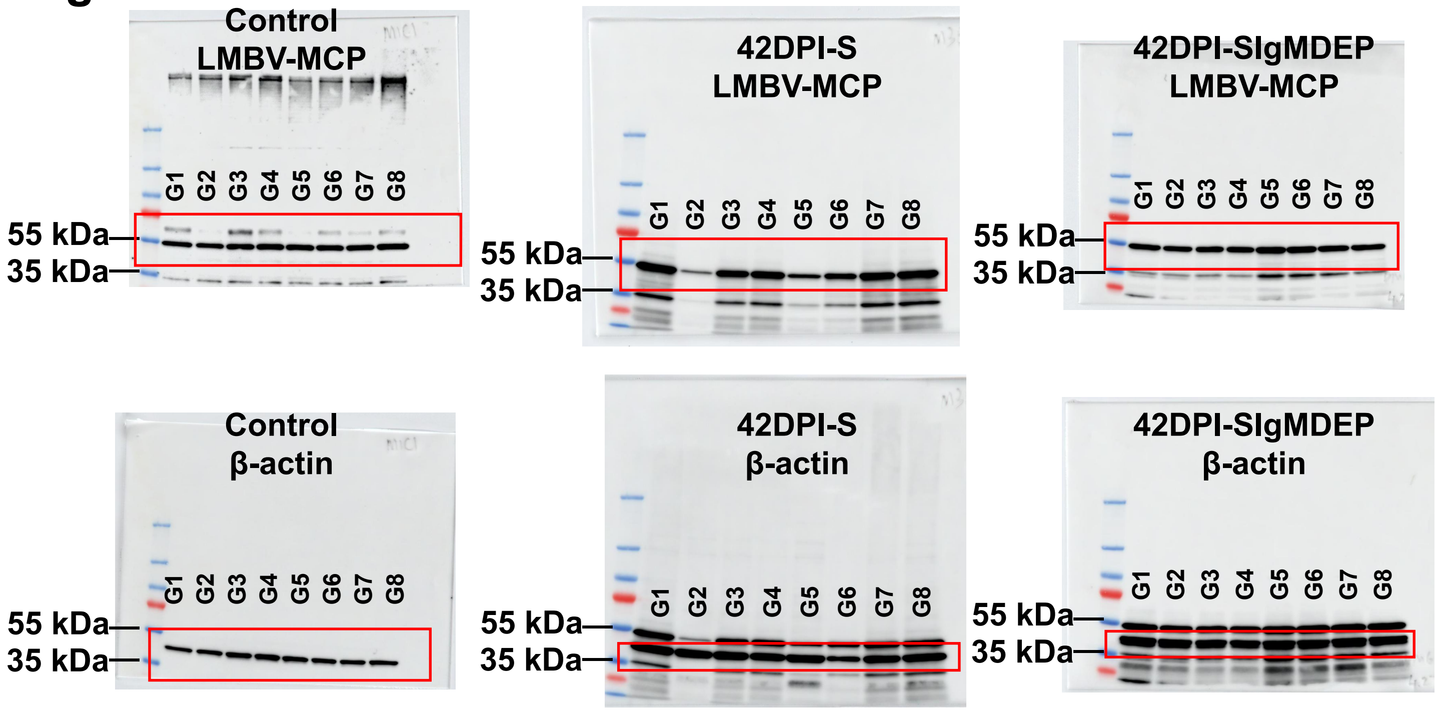

Figure 7-source data 1 Original membranes corresponding to Figure 7D and 7F. In Figure 7D (upper panel) and Figure 7F (lower panel), the first row of membranes corresponds to LMBV-MCP, and the second row of membranes corresponds to β-actin.Rainbow molecular weight markers were employed.The areas highlighted by red boxes are used in this result figure.
