# Supplementary material for: Newly discovered and conserved role of IgM against viral infection in an early vertebrate
Source: eLife. 2025 Sep 4;14:RP104465. doi: 10.7554/eLife.104465 (PMC12410970; doi:10.7554/eLife.104465)
Supplement: Supplementary file 1. [file elife-104465-supp1.docx]

| **Gene** | **GenBank**  **accession no.** | **Primer Sequence (5’-> 3’)** | |
| --- | --- | --- | --- |
|  |  | **Forward primer** | **Reverse primer** |
| IRF-3 | XM_038735465.1 | TCTCATCTTTAAGGCGTGGGC | GGGGTTAGCGGTGTCGTTC |
| IRF-7 | XM_038706685.1 | GCTGCCACTGTGTTTGTC | CTGTCCTTTCCCTTATGC |
| TRIM25 | XM_038714419.1 | GTCACCAGTCACCACGCTTTC | CCACTTGGCTTATTTCCTCCC |
| IFN-γ | XM_046040264.1 | TCCCTCTGAAGATGAACAAA | AACGCCACCCATAAACA |
| MX1 | XM_046075478.1 | TCTACGATGAAGGCCACGCTA | TCTTCCACGGTACTTTCGTTC |
| DHX-58 | XM_038704586.1 | GACTGATGCTTATCGCTCCCTA | ACGCTTTTACGGGTTTTACTGA |
| IL-1β | XM_038733429.1 | CGTACATCCGTGCCAACAGT | ATGCTCTTTAACTCCTCCT |
| IL-6 | XM_046059156.1 | GGGAGACTCGCTCTGACCTACTG | TACCTCCTCCTTGTGGCGTTGG |
| IL-8 | XM_038704090.1 | GAGCCATTTTTCCTGGTGACT | TCCTCATTGGTGCTGAAAGATC |
| IL-10 | XM_038696252.1 | CGGCACAGAAATCCCAGAGC | CAGCAGGCTCACAAAATAAACATCT |
| TGF-β | XM_038710299.1 | ACAACTTCATCCATGGCGGT | TCGTTGATTCTTGGGCTGGG |
| TNF-α | [XM_038723994.1](https://www.ncbi.nlm.nih.gov/nucleotide/XM_038723994.1?report=genbank&log$=nuclalign&blast_rank=3&RID=A63EAH14013) | CAGTGCAATGGCAGAACCAG | TTGACCCTGAAGGACGCTTG |
| IgM | MN871984.1 | GTTACCTTCTCCTGCTTG | GTTCCGTTCTCATAGTTTC |
| IgT | MZ388129.1 | GAAGGTCAACAACGCTGAGTG | TGTTGCTGGTCACATCTAGTCC |
| IgD | MZ388128.1 | AAGGGAAACAGTGCTGTGCT | TTGCCAGTGGGGTTTGACTT |
| β-actin | XM_038708956.1 | CAACGATATCCGTCGTGGCA | ACAGCGAAACGACCAAGAGG |
